# Autonomous Vehicles Enabled by the Integration of IoT, Edge Intelligence, 5G, and Blockchain

**DOI:** 10.3390/s23041963

**Published:** 2023-02-09

**Authors:** Anushka Biswas, Hwang-Cheng Wang

**Affiliations:** 1 Department of Power Engineering, Jadavpur University, Kolkata 700056, India; 2 Department of Electronic Engineering, National Ilan University, Yilan 260007, Taiwan

**Keywords:** autonomous vehicles, Internet of Things, Internet of Vehicles, Internet of Autonomous Vehicles, artificial intelligence, edge computing, edge intelligence, vehicle to everything, 5G, 6G, blockchain

## Abstract

The wave of modernization around us has put the automotive industry on the brink of a paradigm shift. Leveraging the ever-evolving technologies, vehicles are steadily transitioning towards automated driving to constitute an integral part of the intelligent transportation system (ITS). The term autonomous vehicle has become ubiquitous in our lives, owing to the extensive research and development that frequently make headlines. Nonetheless, the flourishing of AVs hinges on many factors due to the extremely stringent demands for safety, security, and reliability. Cutting-edge technologies play critical roles in tackling complicated issues. Assimilating trailblazing technologies such as the Internet of Things (IoT), edge intelligence (EI), 5G, and Blockchain into the AV architecture will unlock the potential of an efficient and sustainable transportation system. This paper provides a comprehensive review of the state-of-the-art in the literature on the impact and implementation of the aforementioned technologies into AV architectures, along with the challenges faced by each of them. We also provide insights into the technological offshoots concerning their seamless integration to fulfill the requirements of AVs. Finally, the paper sheds light on future research directions and opportunities that will spur further developments. Exploring the integration of key enabling technologies in a single work will serve as a valuable reference for the community interested in the relevant issues surrounding AV research.

## 1. Introduction

Intelligent transportation systems (ITSs) are systems that use various automation technologies, computers, controls, and communication to enhance the safety and efficiency of transportation, in addition to being more energy-efficient and environmentally friendly. Among the various scopes of ITSs, road surface transportation is heavily emphasized, in which autonomous vehicles (AVs) are the most integral part. AVs incorporate a wide range of diverse technologies pertaining to electronics, vehicle dynamics, communications, control, sensing, and proper knowledge of human behavioral instincts on the road. In a matter of years, existing human-controlled automobiles will be viewed only as a remnant of our past, as AVs are gradually revving up to rule the roads. After decades of regular research spurred by a precipitous increase in investments made by technological giants across the world, we are now ready to enter the AV era. Clasping the hands of the Internet of Things, edge intelligence, and being buoyed by new advancements in blockchain smart contracts and vehicular networks, AVs will be equipped with a number of desirable properties such as self-verifying, self-executing, immutability, data reliability, and confidentiality that will cater to every need of the users. With the potential to solve transportation-related economic and environmental issues, reduce road congestion and accidents, and lower emission levels, AVs have emerged as the most-promising solution among all. AVs can mitigate accidents caused by human errors such as speeding, and distracted driving due to the effect of alcohol or other substances, which account for an alarming 93% of the total number of crashes [1] by minimizing the involvement of human drivers. According to the Insurance Institute for Highway Safety [2], partially autonomous system features such as forward collision avoidance, lane departure warnings, and side view assist, among others, can avert crashes and reduce injuries and fatalities by as much as 33%! Among a long list of advantages, including fuel savings, better fleet management, better commuting experience, and improved traffic conditions, AVs can also enhance mobility for disabled people and those that are too young or too old to drive, by enabling them to explore their independence and improve their social life [3,4]. However, level 5 autonomous vehicles are still farther from reality due to several technological barriers, besides trust and safety issues, which are being intensely researched upon. With the on-road testing of AVs by technological giants such as Google, Tesla, Audi, BMW, Mercedes-Benz, and others aiding the current research regarding the major bottlenecks of the AV architecture, an updated statistical point of view is still lacking [5]. For the quicker commercialization of AVs, more insights are desirable to make them a safer and more trustworthy option for people.

Autonomous vehicles are no longer a faraway vision, with some current instances developed by Tesla, Google, and their rivals incorporating some self-driving features. The Internet of Things (IoT) has been one of the driving forces behind the actualization of our AV dream. The Internet of Vehicles (IoV), born from IoT and soon to transform into the Internet of Autonomous Vehicles (IoAV), will transform a vehicle into an intelligent agent in local collaboration with its neighbors for content sharing [6] that will assist and ultimately take over control from human drivers in the near future. As more and more devices are coming under the umbrella of an enormous connected network and, thereby, becoming ‘smarter’, thanks to precipitous advancements in IoT, the data traffic arising due to their interactions is also growing exponentially. With such a gigantic amount of data floating around in various clouds, we cannot expect low latency, a quick response time, and excellent quality of service (QoS). Fog computing, mobile cloud computing (MCC), and edge computing have solved this issue to a great extent, but they are still riddled with infrastructural and security issues. Our vision of having fully autonomous cars gracing the roads was brought within our grasp by the boom in the artificial intelligence (AI) domain. Due to the functional necessity of AI to analyze huge volumes of data and extract insights, integrating AI with edge computing is seen as a potential solution, giving rise to edge intelligence (EI). It is not merely a simple collaboration between them but involves the complex amalgamation of several technologies and concepts. This includes, but is not limited to, intelligent offloading to edge servers, intelligent collaboration among edge servers, and analyzing data locally at the edge (from where the data originate) instead of the cloud ushering in a world with over trillions of smart IoT devices [7,8]. To achieve full autonomy over vehicular control, the vehicle needs to touch or even exceed human perception, decision making, and intelligence, which can be achieved with stronger AI and ML algorithms coupled with efficient vehicle-to-everything (V2X) communications [9]. V2X as a complementary technology, providing a 360-degree environmental awareness to the vehicle, will be buoyed by the arrival of 5G and 6G communication technologies, which aim to deliver ultra-reliable and ultra-low latency transmissions for smooth vehicular communications. The complexity of the AV infrastructure makes it vulnerable to security and privacy attacks that can endanger the lives of passengers and commuters. Surpassing traditional centralized security systems, blockchain has emerged as the best solution to provide the much-needed security shield to AVs with its data transparency, immutability, and decentralized approach. Therefore, enriching and enmeshing the aforementioned technologies into a robust system will lead to the realization of our much-yearned AV dream into reality. This paper attempts to bring them together and provide a comprehensive state-of-the-art review of the important topics at hand.

## 2. Contributions of the Paper

Most of the works surrounding AVs and their supporting technologies focus on one technology or just a part of it. In other words, they are very specific. To the best of our knowledge, a paper covering the key technologies related to AV and their related technological offshoots for smooth integration into the AV architecture is missing. The uniqueness of this work lies in fulfilling the aforementioned need to have a comprehensive review that will provide insights into the pillars that support AVs. Before venturing into any specific part of the aforementioned technologies needed for autonomous vehicles, readers will have sound knowledge on the other technologies supporting the AV architecture, besides their area of interest. By referring to some among the plethora of references cited in this work, gaining further insights will become easier.

Compared with the existing works surrounding autonomous vehicles, the main contributions of the paper are summarized as follows:

1. A thorough state-of-the-art analysis of four main technologies, namely IoT, edge intelligence, 5G and 6G communications and blockchain in relation to AVs has been performed in a single work. This will provide a good basis for understanding the technologies that govern the functioning of an autonomous vehicle. Thus, readers will have sound knowledge of the itineraries of these before deciding to conduct further research into any of these domains or those under its umbrella associated with AVs.
2. It attempts to shed light on why these technologies are indispensable for supporting the AV framework by discussing them in detail: right from their basic introduction to the technical essentials to construct a suitable platform based on the respective technologies which are needed for boosting the AV architecture.
3. Besides listing the significant barriers that are currently curtailing the further advancement and commercialization of the autonomous vehicle industry, we have also enumerated the myriad challenges associated with each of these technologies separately that are preventing their effortless integration into the AV infrastructure. These, along with the future research directions at the end, will spur extensive discussions in the community and inspire further research to advance towards the AV dream.
4. Through discussions on edge intelligence, a relatively new domain that requires extensive research like its other contemporary, more-developed fields, our paper will be one among the limited works at the frontiers that depict edge intelligence as a suitable platform for autonomous vehicles. In the respective section, edge intelligence has been detailed thoroughly by first discussing its building blocks: artificial intelligence and edge computing platforms concerning autonomous vehicles. Under that framework, the integration of artificial intelligence and edge computing has been explored by discussing the architectures and technologies needed for distributed training at the edge and those essential for model inference. This enhances the uniqueness of our work by deliberating upon edge intelligence, first through examining its building blocks (AI and edge computing) and then discussing their combined infrastructure to be further developed for AVs.
5. After enumerating the main constituents or building blocks of 5G needed for vehicle-to-everything (V2X) communications that are so crucial to AVs, such as proximity service, mobile edge computing, and network slicing, the paper has provided a brief yet informative introduction to 6G communication that has gained significant popularity in recent times.
6. The section on blockchain discusses its important layers that are crucial to the architecture needed for AVs or other applications. Apart from that, some of the main security issues pertaining to blockchain are also detailed.

### Organization of the Paper

**Figure 1:**
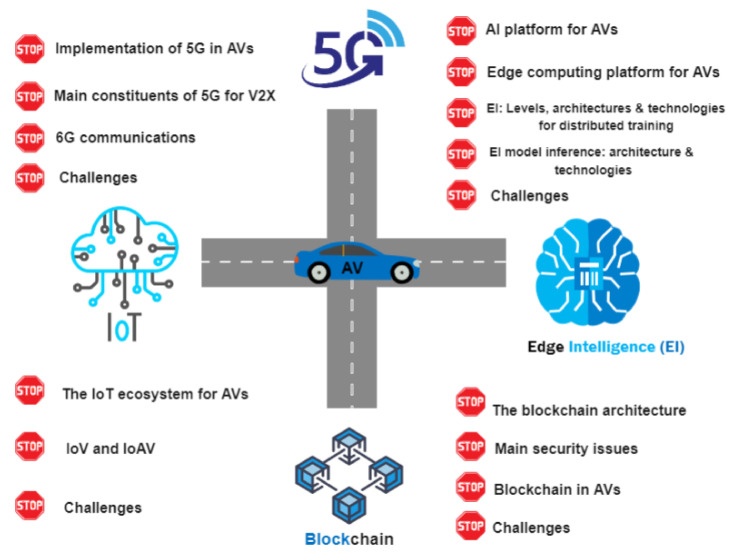
Enabling technologies for autonomous vehicles. The figure shows the four technologies which have been discussed at length in the paper, specifically in the context of autonomous vehicles. The multiple "stop" signs signify the various sub-sections where readers will have to stop and delve further. This is an attempt to graphically (and literally) represent the road-map followed in the paper.

The rest of the paper is arranged as follows. Section 2 lists the six levels of automation, as laid down by the Society of Automotive Engineers (SAE). Section 3 sheds light on some of the significant current challenges being faced by autonomous vehicles, including the technological barriers and the legal and ethical issues. Then, we review each of the four technologies, commencing with a brief introduction to the Internet of Things (IoT), and then moving on to the IoT platform for AV, IoV, and IoAV, and some challenges specific to it in Section 4. Section 5 introduces edge intelligence (EI) as a potential platform for autonomous vehicles. In the corresponding subsections, the two main constituents of EI, namely artificial intelligence (AI) and edge computing, are discussed with respect to their roles in the functioning of AV. Then, two separate subsections are dedicated to the levels of EI and the architecture and technologies essential for distributed training and model inference, followed by the main challenges of EI, AI, and edge computing in the last subsection. Section 6 explores the 5G technology in the context of AVs, the ways of implementing it along with other technologies in AV, some building blocks of 5G for V2X, an introduction to 6G as the rising successor, and the main barriers involved with 5G. Last but not least, blockchain forms the subject of Section 8, with a description of its layers, security issues, applications in AVs, and further challenges. Some notable future research directions for AV infrastructure are presented in Section 9, which will spur further discussions and promote new research. Section 10 concludes this paper. Figure 1 graphically depicts the four enabling technologies surrounding autonomous vehicles.

## 3. The Six Levels of Automation

With autonomous vehicles quickly gaining popularity across the world, in order to assess how close we are to realizing our AV dream, in reality, we need to compare the performance of an AV’s dynamic driving task (DDT) with a detailed hierarchical table. SAE provides a detailed taxonomy of the six levels of automation (Level 0–Level 5). They are as follows [10]:

- Level 0 (no automation): The majority of the vehicles found on the road belong to this level. They are fully controlled by humans who provide the DDT. Nevertheless, there may be systems to momentarily assist the driver in case of an emergency. Since they do not drive the car without human intervention, those emergency systems cannot be accepted as a type of automation.
- Level 1 (driver assistance): This is the lowest level of automation. The vehicle may incorporate systems to assist the driver temporarily in specific situations. Adaptive cruise control, in which the vehicle can stay at a safe distance from its preceding car, can be accepted as Level 1 because it allows (and in a way, assists) the human driver to focus on the other nuances of driving, including steering and braking.
- Level 2 (partial driving automation): Level 2 is also known as an advanced driver assistance system (ADAS), in which the vehicle can control both the steering and speed, though the presence of a human driver is necessary at the steering wheel. This is why it falls short of being called ‘self-driving’ because humans can take over vehicular control anytime. Tesla Autopilot qualifies as Level 2.
- Level 3 (conditional driving automation): The vehicle has gained more autonomy than Level 2 vehicles. These are equipped with many sensors to detect the environment and make informed decisions on their own. However, an alert human driver that is able to take over the control during any unexpected situation when the system may fail is still required. Automated emergency braking (AEB), driver monitoring (DM), and traffic jam assist (TJA) are some extra functionalities other than those already present in Level 2 vehicles.
- Level 4 (high driving automation): Level 4 vehicles can intervene in case things go wrong or in case of system failure. This differentiates Level 4 from Level 3 vehicles. These can operate in self-driving mode but for now, are confined only to a limited low-speed urban area (known as geo-fencing). This is because the legislation and infrastructure associated with it have to evolve further. These do not require human intervention under most circumstances, but a human driver can still choose to be present and take control anytime.
- Level 5 (full driving automation): This is the pinnacle of the autonomous vehicle dream. Level 5 vehicles do not require human intervention at all, and the dynamic driving task is eliminated altogether. Even the steering wheel and acceleration/braking pedals will be absent. These will be able to maneuver the vehicle in all the ways a typical human driver can. A level 5 vehicle will be free from geo-fencing and can go anywhere on its own. Currently, a lot of extensive research and testing concerning Level 5 vehicles are going on in different parts of the world, but it will take some more time before they are available to the general public.

**Figure 2:**
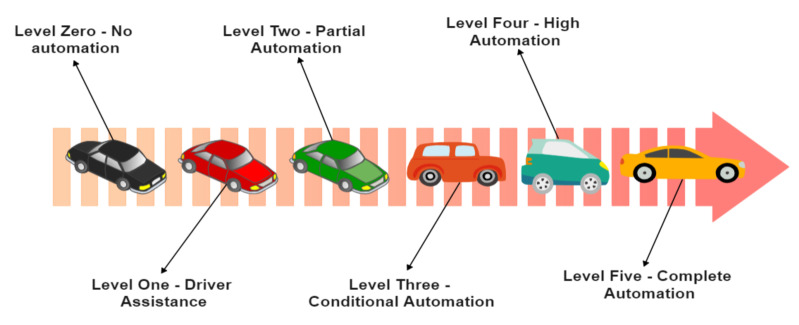
The six levels of driving automation.

The six levels of driving automation are illustrated in Figure 2.

## 4. Current Challenges

Despite the current accelerated and intensive research into the field of autonomous vehicles to bring them to the roads, the field is still riddled with technical [11], legal, and moral challenges. Unless the following obstacles are eliminated or reduced to a considerable extent, it would not be safe or ethical to launch AVs in the market.

### 4.1. Infeasible Sensing

**Figure 3:**
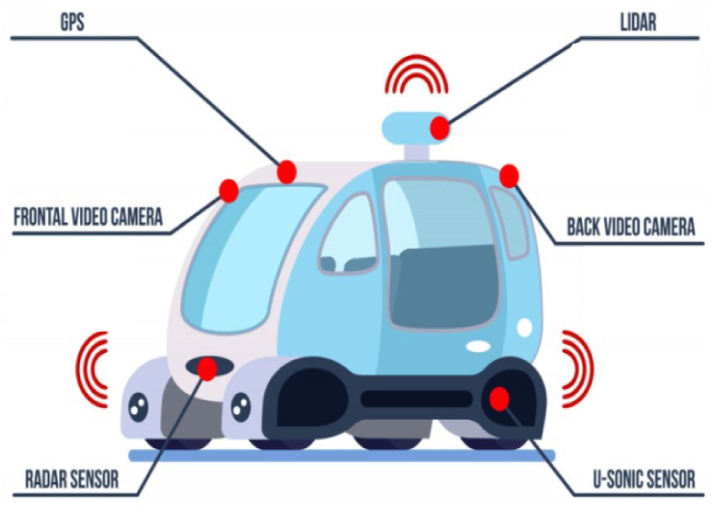
The main set of sensors present in an AV, utilized in sensor fusion.

For the safe journey of an AV, the sub-task of object detection is one of the most important prerequisites, as it allows the car controller to account for various obstacles while considering possible future trajectories. Sensing the surroundings of the AV heavily depends on the intrinsic properties of the embedded sensors and their quality of perception. Figure 3 shows the multiple sensors present in an AV to efficiently sense their surroundings. The four main types of sensors are [12]:

- GNSS/IMU: global navigation satellite system and the inertial measurement unit are used for the localization of the AV. GNSS reports the global position estimate accurately, but its update rate is too slow to match real-time requirements. IMU reports the inertial updates of a body at high frequency (at or higher than 200 Hz) but its accuracy degrades with time. Kalman filtering is used to obtain the best advantages of the two, which gives the vehicle a dead reckoning capability and the ability to gather accurate data for real-time localization [13].
- LiDAR: Light detection and ranging (LiDAR) can be used for scanning, localization, obstacle detection, and accurate depth perception. It works by calculating how long it takes for a beam of light to hit surfaces and bounce back to the laser scanner. The distance is computed using the velocity of light. This approach is known as “time of flight” measurements. The higher the number of layers used in the scanner, the better the perception of the wide range of environmental contours [14]. They generate a "shape" of the surrounding environment in the form of point clouds. Consequently, a particle filter is used to filter out and compare the observed shape against a map of known objects to reduce ambiguity.
- Cameras: Monocular cameras are the most common and widely used sensors in an AV, capturing the 2D RGB images of the surroundings, used for object recognition, tracking, traffic light detection, etc. AVs usually mount between eight and ten cameras, running at approximately 60 Hz and together generating a high amount of data per second. To add depth perception, many systems use double-lens (binocular) cameras called stereo cameras [15]. Both the single lens and the stereo cameras are cheap, allowing them to be used and depended upon extensively.
- RaDAR: RaDAR measures distances, movements, and velocity by sending out Radio waves that reflect back from obstacles and detect short- and long-range depth. Short-range Radars have a range of 20–50 m, whereas long-range radars can extend up to 250 m. Radio waves can penetrate objects and are unaffected by bad weather conditions, unlike LiDAR and cameras. They report the distance of the nearest obstacle and the data generated by them do not require a lot of processing. Hence, they are typically fed as input to the control processor for realizing adaptive cruise control (ACC), blind-spot monitoring (BSM) and predictive emergency braking systems (PEBS) [16].

Table 1 lists the various disadvantages associated with each type of sensor used in AV. Besides those, vehicle computing systems are heavily constrained by memory [17]. Hence, it is not possible to store and run detectors with large volumes of input images which constrain the depth of neural network approaches. There is also the requirement that the entire detection process be fast, which usually leaves no room for image pre-processing to boost detection performance. Maintaining a high level of accuracy with a quick response time is key. One currently used solution is sensor fusion [18], which combines various sensing modalities for perception, such as combining data from LiDAR sensors, radar, sensors atop traffic lights, and sensors in other vehicles. Thus, it improves the accuracy and quality of sensing, in addition to reducing the ambiguities that may come from the use of various sensors. The vehicle positioning and orientation will be calculated by combining data from separate sensors. The fusion of data from different sensors and AI-based intelligent sensing are also extensively discussed in [19]. The types of multi-sensor fusion discussed in various literature [20,21,22] involve camera-radar (CR), camera-LiDAR (CL), and camera-LiDAR-radar (CLR). The paper [23] extensively reviews the sensors used, their pros and cons, and the various sensor fusion technology approaches adopted in AVs.

### 4.2. Clash between Reliability and Latency

Latency refers to the time for a data packet to be transmitted and processed through multiple intermediate devices and eventually arrive at the destination and be decoded. Besides considering the inference accuracy, we should also pay some attention to another important aspect, namely inference delay. It is obvious that the quality of data and shallow neural networks can significantly hamper the inference accuracy in a pre-trained deep learning model. For the data captured by the sensors on the AV to be reliable and worthy of the decisions based on them, there should be some room for the preprocessing of data to accentuate its quality and hence, allow the vehicle to make correct and ethical decisions at the right moments. However, an additional communication delay is introduced to account for the time taken for data offloading from the mobile devices to a more powerful edge server. Sometimes it may become hampered by the channel dynamics. Nevertheless, the delay introduced by all these vastly endangers the reliability of autonomous vehicles, since even a delay of a few milliseconds can turn out to be fatal while sensing and deciding how to overcome an obstacle on road, which can even cost the lives of pedestrians and other commuters. Therefore, to achieve a comfortable balance between reliability and incurred latency, it is of utmost importance to cut down the wireless transmission delay between the devices on-board and the edge server.

### 4.3. Limited Resources

Unlike the cloud servers which have a large number of powerful graphics processing units (GPUs) and central processing units (CPUs), the edge servers are not as heavily equipped [30] in consideration of the economic benefits and scalability of deployment. For instance, there are a plethora of edge servers that are deployed close to users. As a result, the economic factors of such large-scale deployment automatically come into play. Therefore, an edge server does not need and cannot have as many resources as a cloud server. Thus, they can hardly take a massive number of offloading requests from mobile devices due to constraints in memory, computing, data caching, power resources, limited communication bandwidth, and ultimately, may not be able to process all the tasks fully. If all the data are indiscriminately offloaded to the edge servers, this will lower the processing efficiency and increase the latency of the network.

### 4.4. Cyber Security and Privacy

The data acquired by AVs for processing and inferencing are always exposed to a number of security threats due to unauthorized access and weak protection against malicious entities that may jeopardize the private information of the owner. Cybersecurity and privacy are two of the leading bottlenecks hindering the wide deployment of AVs and public acceptance. A survey conducted in 2015 with 5000 respondents across 109 countries [31] revealed people’s wariness and concerns regarding the misuse of personal information through the software hacking of vehicles with all levels of automation. The situation had not improved much by 2022, as the main threats had not been eliminated. Cybersecurity is the main liability hazard arising from loopholes in in-vehicle security systems such as fragile connectivity, open channels, insecure bus systems, and the existence of intelligent hackers. The hardware and software systems of an AV can be compromised in the following ways [32]:

- Intelligent hackers can take over the AV and connected vehicles through their wireless networks (Bluetooth, cellular networks, etc.) to sell personal information for financial gains, inflict physical harm or carry out unlawful activities such as drug and human trafficking. This is relatively easier, as demonstrated by a study [33] wherein they took control of the brakes and engines of a Chrysler Jeep by hacking its Internet connection.
- GNSS data can be remotely manipulated to create confusion or critically endanger passenger safety. This can be achieved by injecting fake messages or spoofing GNSS [34].
- Physical attacks on sensors include the use of bright lights to blind cameras, and creating interference using ultrasound or radio waves to distract other sensors from correctly perceiving obstacles. Such situations may even lead to fatal accidents. Other onboard hardware may be tampered with leading to privacy breaches.
- The attacker may even intercept messages in intra-vehicle and inter-vehicle communication (V2V and V2I) and gravely endanger the safety and privacy of the owners and other AVs.

Cyberattacks [35,36] can lead to functional safety issues and can easily lead to privacy and/or identity theft, even costing someone’s life, if the attacker deliberately changes the direction and takes full control over the actions of the AV. This has prompted companies and governments to take precautionary steps. Various software may be installed to detect malfunction or the presence of hackers, with frequent software updates and changing security architectures. Governments in the US, China, EU, and Singapore have enacted new legislation to address privacy and cybersecurity risks along with the adoption of a control-oriented strategy. Stronger laws and better software can go a long way in tackling these issues.

### 4.5. Legal Issues

As autonomous vehicles gradually take over driving control, the law must alter its code and implementation. Worldwide regulations exist to provide the safest and the most secure travel experience to people. Therefore, autonomous vehicles must prove that they conform to the desired safety standard. Current research in the US and Europe is working on this [37,38]. Legal challenges are one of the most critical issues concerning AVs, covering myriad public policies, traffic codes, technical standards of conventional vehicles, and tort law [39]. The use of the term “autonomous” in the case of vehicles has sometimes been misconstrued by the law because “autonomy” has broader philosophical connotations, unlike the technical one, which simply means that it can work independently of human intervention while driving [40]. The Convention of Road Traffic of many countries still mandatorily requires the presence of a driver who shall, at times, be able to take control of the vehicle. This provides a legal framework for semi-autonomous vehicles, but the fully autonomous ones are still off the hook, for which they need to prove that they are either safer than or as safe as their predecessors.

### 4.6. Moral and Ethical Issues

When faced with unexpected traffic situations that require complex decision making within split seconds, human drivers are not expected to react optimally and may be excused for making wrong decisions. However, for AVs, which are capable of analyzing the potential outcomes of various options and taking actions accordingly within milli-seconds, wrongful decision making then becomes part of extensive debate and legislation. The AV must conform to the expected moral norms, which differ from person to person. For instance, personality traits determine whether the driver would like to endanger their own life to save others [41]. It was found in a study [42] that participants programming an AV tend to more readily endanger car occupants than pedestrians compared to participants driving in a simulator. There is growing evidence of discrepancies between moral judgments (what they would do in moral dilemmas) and moral action (what they would actually do) [43,44]. What is considered ethical for human drivers may not be so for self-driving cars, and the evaluation of morality may vary based on the perspective of the way that the situation has been presented. Would it be acceptable that, because of AVs, fewer people are harmed, but pedestrians become the ones more likely to be harmed than vehicle passengers? Thus, the introduction of AVs may put different groups at risk compared to the current situation.

## 5. Internet of Things

Currently, there is a lot of buzz around the technical term “Internet of Things” (IoT), which is said to have been suggested by Kevin Ashton as early as 1999 [45]. In recent few years, many varied definitions of IoT have sprung up and are being used everywhere. For instance, the International Telecommunications Union defines the Internet of Things as ‘‘a global infrastructure for the Information Society, enabling advanced services by interconnecting (physical and virtual) things based on existing and evolving, interoperable information and communication technologies’’ [46]. In a nutshell, IoT is a giant network of interconnected things and people, all of which collect and share data about the way they are used and about the environment around them. Although the meaning of “things” has evolved considerably over the last decade, the main goal for a device to make sense of the information without human intervention remains largely unchanged. Machine-to-machine (M2M) interaction connects isolated sensor systems to servers with little or no human interaction. IoT takes M2M connectivity, integrates with web applications, and connects to the cloud computing system. Physical things and IT are combined in IoT, in the form of hardware and software, to bring about innovation in ideas and existing models. Therefore, the primary characteristics of the physical “thing” are strengthened with additional IT-based digital services, which can be accessed locally and globally [47]. The areas of application of IoT are diverse and numerous. In the near future, they may as well extend to virtually all frontiers of everyday life. For instance, in the Fourth Industrial Revolution (Industry 4.0), smart industries are more concerned about the development of intelligent production systems and connected production sites. Smart homes, smart transportation systems, and smart healthcare systems are some other major areas of the application of IoT. A detailed study on the impact of IoT in developing smart cities was analyzed in [48].

### 5.1. The IoT Ecosystem for Autonomous Vehicles

Systems wherein computing, communication, and control technologies are tightly linked are known as cyber-physical systems (CPSs). CPSs, which collect data from sensor networks to be processed in real-time, will drastically alter our future. Intelligent transportation systems will be born out of these, in which most of the tasks (navigation, decision making, and so on) will be controlled by the vehicles themselves with no human intervention. IoT solutions that facilitate communication between vehicles are the building blocks of ITSs. The ITS–IoT system forms an ecosystem with sensor systems, monitoring systems, and display systems. A detailed architecture of such an ecosystem has been proposed in [49].

**Figure 4:**
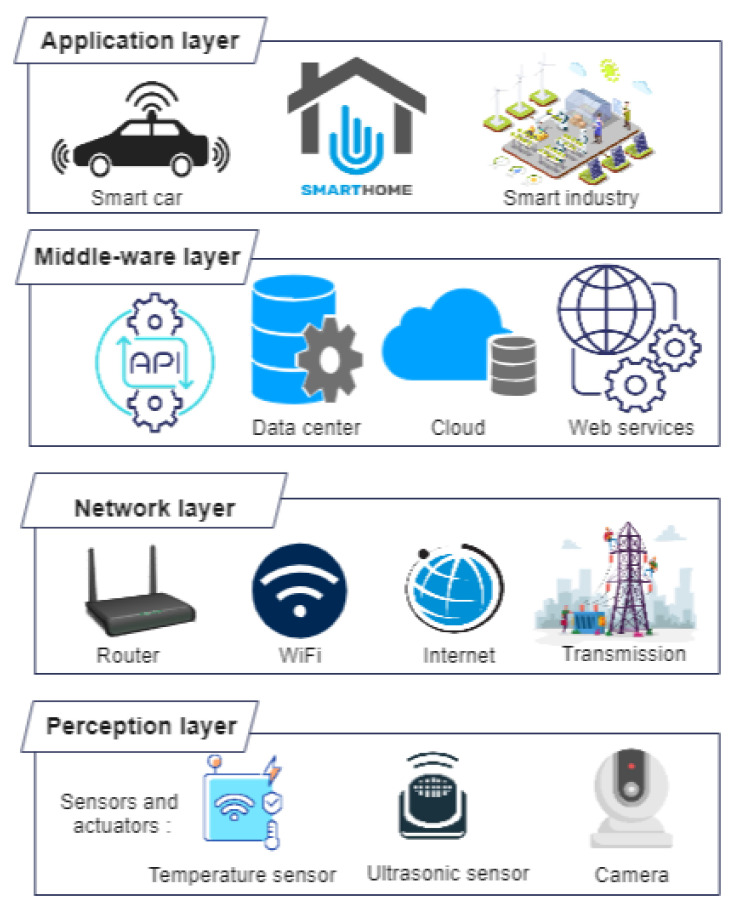
Layers of an IoT system.

Figure 4 shows that an IoT platform has four core layers [47]:

- Perception layer: also known as the thing or device layer, it comprises all the necessary hardware including sensors, actuators, processors, and embedded software, which collect diverse data from the physical world.
- Network layer: the connectivity layer or communication network layer has routers and gateways. It includes all the wireless technologies such as Wi-Fi and cellular technologies including 4G or 5G, 6G, and communication protocols such as MQTT, that facilitate the communication between the device layer and the cloud layer.
- Processing layer: acting as the middleware, this layer is responsible for processing the data received from its preceding layers. For non-real-time applications, real-time data are captured using APIs and put to rest. This layer is important since it decides the utility of the processed data in terms of user requirements and where it should be placed next. It sorts un-categorized data and deals with its accessibility to other layers, devices, or systems. In this way, it enhances the interoperability of IoT devices.
- Application layer: also called the IoT cloud layer, it is the topmost layer that contains important servers or clouds for storage and analysis of data. Furthermore, device communication and management software is used to communicate with people, systems, and things, thereby managing the “connected things". It acts as a centralized management system and answers prominent business questions.

Besides the aforementioned, primary layers, two additional layers exist encompassing them. These are:

- Edge or fog computing layer: this extra layer helps IoT devices to meet the latency, speed, and accuracy requisites of 5G and beyond 5G communication networks. Processing and storing information early and close to its origin (edge or fog nodes) helps to conserve computation resources, save time, and enhance system performance.
- Security layer: It acts like a cap over all the preceding layers to prevent security breaches. Security can be compromised by malicious entities at any of the aforementioned layers, during transmission through the network, or at the end devices. Therefore, it deals with device security, connection security, and cloud security.

An IoT ecosystem comprises six components that interact among themselves: vehicle, person, personal device, network infrastructure, sensing device, and roadside device. Their significance is as follows [50]:

- There will be a connected framework of autonomous vehicles in the neighborhood, which will communicate among themselves to share relevant information, such as road conditions, alerts, and other physical parameters.
- Person includes anyone who requests access to a service in the IoT ecosystem. It may be a fellow commuter, pedestrian, or people living in the neighborhood.
- Personal device belongs to any of the aforementioned categories of people and uses or provides services.
- Network infrastructure devices in the communication loop are those used to transfer data to the ecosystem.
- Sensing devices are the embedded sensors and actuators in the AVs that collect and transmit information about vehicular parameters (temperature, tire pressure, fuel consumed and remaining fuel), a person’s health (blood pressure, heart rate) and environmental factors (noise level, weather conditions, pollution, etc.).
- Roadside devices form the transportation environment (information screens, traffic lights, or radars) that catch and disseminate necessary information to the ecosystem about road conditions, possible detours, accidents, etc.

In such an expansive and comprehensive IoT ecosystem, there is a multi-level data exchange among all the connected devices that are constitute part of it. Such device-to-device (D2D) interactions can include devices inside and outside the vehicle, which collect data, store, process, communicate them, and make decisions based on them with no human intervention. Six types of D2D interactions, as detailed in [51], are vehicle-to-vehicle (V2V), vehicle and personal device (V2P) (or vehicle-to-human devices, V2H), vehicle and roadside (V2R), vehicle and sensor (V2S), vehicle and infrastructure (V2I), and roadside and personal device (R2P). Two more interactions, namely roadside-to-roadside (R2R) and sensors and actuators (S2A), can also be included in the above list.

### 5.2. IoV and IoAV

The rise and subsequent expansion of IoT technology have led to extensive research in application-specific areas. Vehicular ad hoc networks (VANETs) are one of them, which throws light on inter-vehicular communication and communication with the infrastructure [52]. Over time, VANET has evolved into the IoV, a direct offshoot of the IoT, and will evolve into the Internet of Autonomous Vehicles (IoAV) [53]. As suggested by the name itself, IoV is all about collecting data from the on-board sensors of the vehicles and on roadside units, such as GPS modules, proximity radar gyroscopes, LiDARs, and various other performance and control modules present in the on-board unit (OBU). Thereafter, the collected data are processed and can be used alongside similar data (location, speed, traffic, possible detours) about the vehicles from the OBUs of other vehicles to provide a live picture of the traffic for the locality that can better manage the fleet of vehicles [54].

**Figure 5:**
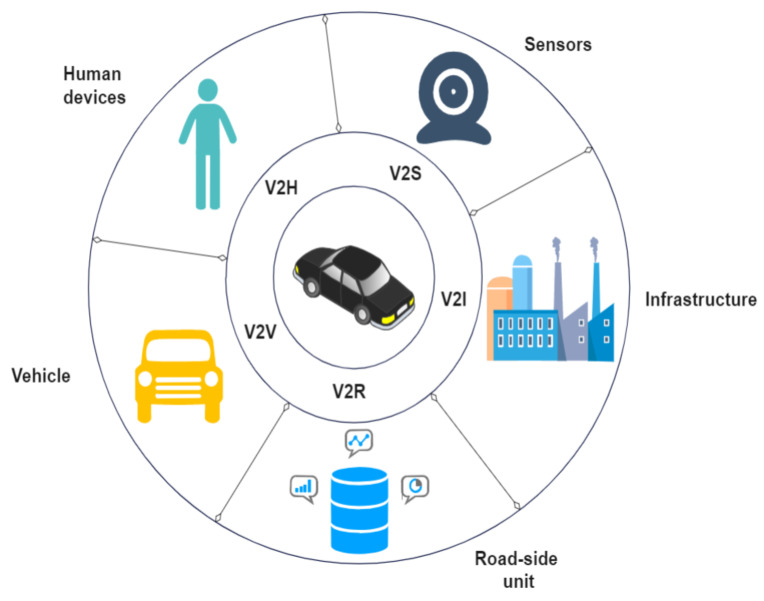
Types of vehicle-to-everything (V2X) communications.

The widely accepted architecture of IoAV comprises three layers: the sensing layer (a combination of physical and data link layers that focuses on collecting information through the available sensors), the network layer, as shown in Figure 5 (handling all types of communication like V2V, V2I, V2P/V2H, V2S, V2R and processing the data using network technologies such as LAN and a transmission medium such as Wi-Fi or Bluetooth), and the application layer (data management, storage, processing, decision making, providing support to big data analysis, WSNs, cloud computing, etc.) [54,55]. In the pioneering work [56], the authors contrasted the difference between IoV and IoAV in terms of tracking behavior, situation awareness, data flow, decision analytics, process optimization, and resource consumption. Social and technical issues that have to be resolved before the widespread adoption of IoAV are also explored.

### 5.3. Challenges

Since IoV and IoAV have evolved from the IoT, they face the same threats as a typical IoT network and encounter a few additional threats, classified roughly into inter-vehicle threats and intra-vehicle threats [53,54,57] such as:

- Security and privacy: IoAV involves sharing the personal information of the user, such as location and identity to collect accurate results. The vehicles in an IoAV network also share information among themselves that may fall into the wrong hands or be manipulated by malicious entities. Since IoV is to be accessed by many devices, it incorporates various technologies and services, which make it vulnerable to DDoS attacks and other malicious risks. Various parts of the AV, such as cameras, GPS, sensors, brakes, alarms, steering wheel, and accelerator, can be remotely accessed and jeopardize the privacy of the users and can even be fatal.
- Real-time response: one major condition for the effortless functioning of an IoAV network is to receive the necessary information and make appropriate decisions with the fastest communication available, which is still lagging due to the current security frameworks that introduce latency in the network due to an intensive authentication process.
- Data validation: the gigantic amount of data generated by an IoAV network needs to be efficiently collected, processed, and authenticated to prevent false-positive reporting. Malicious entities (user or vehicle) may send false data about the traffic or accidents to the network, resulting in unnecessary confusion and traffic diversion for no reason.
- Reliability: for efficient linkage of IoT with autonomous vehicles, having a stable connection is the main prerequisite. Thus, network bottlenecks, DoS attacks, and malfunctions in communication can significantly hamper the working of the infrastructure. Mobility issues should be eradicated and all nodes should be able to transmit and receive information at all speeds and locations. A malfunctioning or unresponsive hardware may also compromise reliability.
- Jamming: IoAV network is vulnerable to jamming attacks such as data jamming, signal jamming, and GPS jamming. The transmission of large amounts of data to roadside units, due to an unanticipated event or attackers deliberately flooding them with garbage information may lead to data jamming, by over-burdening their processing power.
- Huge amounts of information to process: connected vehicles generating approximately 1 GB of data per second, which is destined to increase as more infrastructure and devices go online and demand connectivity. Although Big Data are a perk for IoV technology, managing the constant flow of data can be challenging for providers. Therefore, insufficient storage and long network delays can hamper cloud computing and pose damage to the system.
- Long production cycle: With the currently available IoT infrastructure at hand, it costs manufacturers a great deal of time to build and introduce a connected vehicle into the market. Although this long production cycle time is destined to reduce with further advancements in the IoT/IoV technology, it will still take months to release a smart car into the market. This inhibition may make manufacturers miss development trends and fail to deliver them in the products.

## 6. Edge Intelligence (EI)

At present, “artificial-intelligence” is no longer a far-fetched dream that was founded as an academic discipline in 1956 or a cooked-up work of fiction, as in Mary Shelly’s Frankenstein which had artificial beings endowed with intelligence. Indeed, AI has now become a ubiquitous word that everyone has heard of and needs no explanation. In the simplest terms, any system that emulates human intelligence and cognitive functions to solve a real-world problem and has the ability to think and act as humans can falls under the umbrella of artificial intelligence. The birth of AI was facilitated by Alan Turing’s legendary work computing machinery and intelligence [58] in 1950 where he asked “Can machines think?” and their renowned “Turing Test” where a human interrogator had to distinguish between the text responses provided by a computer and a human. Although much scrutinized, this test has gone down in history as one of the foremost and important concepts of AI. However, the term “artificial intelligence” was first coined by McCarthy, Minsky, and Shannon at Dartmouth College in the summer of 1956, during the Dartmouth Summer Research Project on AI.

There is quite a distinction between strong and weak AI [59,60]. Strong AI is also known as artificial general intelligence (AGI), which would be at par with humans, and artificial super intelligence (ASI) which might surpass human intelligence and is still a far-away future. Therefore, only the weak AI (also called artificial narrow intelligence, ANI) is of commercial importance now, where AI is trained for and used in specific tasks requiring single human capabilities, such as visual perception, understanding context, probabilistic reasoning, and dealing with complexity [61] and exceeds human efficiency by a wide margin. The term “weak” is a misnomer since it has some robust applications such as Apple Siri, Amazon Alexa, IBM Watson, and autonomous vehicles.

To date, intelligent decision making in AVs is generally incomprehensible to humans, which hinders the adoption of the technology by society. AI systems in self-driving cars must not only make real-time and safe decisions but also explain how those decisions are reached to comply with the law in many jurisdictions. Tackling the issue leads to the development of explainable artificial intelligence (XAI) approaches for autonomous vehicles. [62] proposed an XAI framework that considers the social and legal requirements for the explainability of autonomous driving systems. In consideration of the growing relevance of explainable AI, NIST formulated the four principles of XAI [63], which outline the essential criteria that an AI system must meet in order to qualify as XAI:

- Explanation: this principle stipulates that an AI system is able to provide justification, evidence, or support for each decision it undertakes.
- Meaningful: this principle stipulates that the explanation offered by an AI system must make sense to human users. Furthermore, the explanation offered by the AI system must fit the diverse traits and needs of different users considering the diversity in their needs and experiences.
- Explanation accuracy: According to this principle, an AI system’s explanation must accurately mirror the system’s underlying operations.
- Knowledge limits: the AI systems must recognize situations in which they are not intended to function and for which their solutions would not be trustworthy.

These principles are closely related to AVs and should be adopted as guidelines to shepherd the use of AI in AVs. A more thorough analysis of XAI and its taxonomy is provided in [64]. We expect XAI to play an ever-increasing role in AVs to enhance the transparency and trustworthiness of the decision-making process.

With a precipitous increase in the number of inter-connected devices, it is not feasible to transport the vast data generated by them to the cloud for analysis and storage, no matter how efficient and fast our network is. Network disruptions, poor bandwidth, and the induced latency in the network may further conspire to impair such efforts. Therefore, IT architects shifted the focus from the centralized data center, which is incapable of keeping pace with the real-time data needs, to the literal edge of the infrastructure, close to the location where data are generated. Edge computing refers to a distributed computing paradigm that is deployed close to the location where data are produced. This brings the computing and storing of data to the same level where it is produced (at the network edge) and can improve the latency, bandwidth utilization, response time, and QoS [65]. Of the myriad branches of cloud computing that have developed over the years, several variants can be identified:

- Fog computing—data processed outside the network but in a location close to its origin [66].
- Mobile cloud computing (MCC)—a platform where data processing and storage are implemented in the mobile clouds, instead of pushing them to the smart mobile edge devices [67].
- Cloudlet computing—a scaled-down cloud data center at the edge of the internet that provides powerful computing resources to mobile devices that have high latency constraints [68]. It is a heterogeneous network, having all nearby devices such as mobile phones, laptops, and computers cooperate and form a cloudlet [69].
- Mist computing—uses microcontrollers and microcomputers at the extreme edge of the network to transfer data to the fog nodes and eventually to the cloud [70].
- Mobile edge computing (MEC)—a network architecture defined by the European Telecommunications Standards Institute (ETSI) that provides cloud computing capabilities and an IT service environment at the edge of the cellular network [71,72]. Coupled with AI, MEC has emerged as a rising star in the autonomous vehicle industry.

The thriving needs of autonomous driving have led to the confluence of machine learning or specifically, artificial intelligence and MEC or simply, edge computing, resulting in the advent of edge intelligence (EI) or edge AI to greatly facilitate daily life activities [73,74,75]. EI enables the AV to accurately sense its surroundings by offloading the data to the more powerful edge server co-located at the base station (BS). The large amount of data generated and offloaded to the edge requires robust AI algorithms for accurate processing, which necessitated integrated edge intelligence. Thus, the inference computing of AVs can be greatly improved by deploying an EI model to improve accuracy and latency. Since the research on EI is still in its infancy in both academia and industry, it is riddled with significant barriers associated with communication, computing under limited bandwidth, data security, privacy, and energy consumption [76].

The subsequent subsections focus separately on the itineraries of AI and edge computing platforms for autonomous vehicles. The latter subsections go on to elaborate on the EI architecture required by AVs and the various functionalities it supports, followed by the pertinent challenges.

### 6.1. Artificial Intelligence Platform for Autonomous Vehicles

The automotive AI market is valued at close to USD 11k million by 2025. Systems based on AI algorithms will become a standard in the automotive industry in these two categories [77]:

- Infotainment human–machine interface, comprising speech and gesture recognition, eye tracking, driver monitoring, and language interfaces.
- Advanced driver assistance systems (ADASs), which include autonomous vehicles, camera-based machine vision systems, and engine control units (ECUs).

**Figure 6:**
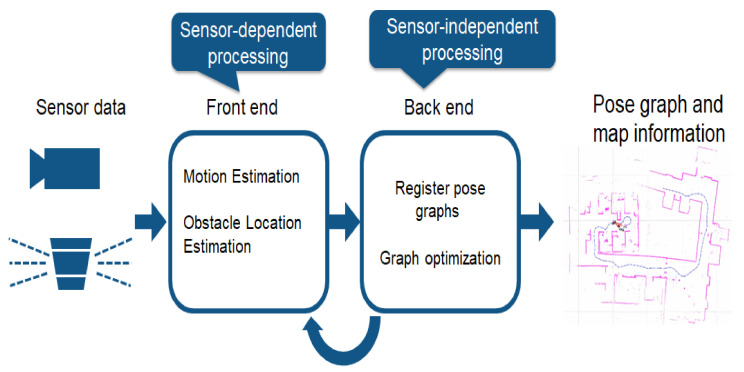
Simultaneous localization and mapping (SLAM). Picture credits: MathWorks.

The advent of deep learning (DL) has helped tackle many challenging AI problems such as appropriate decision making and accurately recognizing obstacles on the road. A lot of papers have forayed into the various ways that AI approaches can provide encouraging solutions to AVs for specific components such as perception [78], motion planning [79], decision making [80], and safety validation [81]. An AI-based AV model has to carry out the following actions [82]:

- Perception: it refers to the AV’s job of continuously scanning and tracking the surrounding environment through various types of available sensors including radar, LiDAR, or cameras, to emulate human vision. The existing perception algorithms available to us can be grouped on the basis of need and the required output, in the following ways:
  - *Mediated perception* uses convolutional neural networks (CNNs) to detect single or multiple images and use them to develop a detailed map of the AV’s surroundings by analyzing their distances from other vehicles, trees, road signs, etc. For instance, AVs can accurately recognize traffic signs using deep neural networks up to an accuracy of 99.46%, which has outperformed human perception in some tests [83]. Other areas such as lane detection and traffic light detection have similar accuracy when using various neural network structures. For instance, YOLO Darknet v2 [84] can detect more than 9000 objects using a CNN model with 40–70 frames per second (fps) in real-time. The accuracy detected by it is an impressive 80% in real-time, which is almost enough to detect most objects in autonomous driving. Novel techniques such as edge detection and salient analysis are implemented in them, to obtain high-definition images of various detected objects. Besides detecting objects, the semantic segmentation of roads with drivable surfaces also falls under the umbrella of AI perception.
  - *Direct perception* provides integrated scene understanding and decision making. AVs create sections of maps (including the distances to other vehicles and lane markings) instead of a detailed local map or a record of trajectory. Thus, direct perception immediately focuses on controlling the steering wheel output and the vehicle speed, while skipping the initial localization and mapping. Papers [85,86] proposed a particular type of CNN framework called PilotNet comprising one normalization layer, five convolutional layers, and three fully connected layers which help AVs steer themselves, with camera and sensor data as input and steering parameters as output.
- Localization and mapping: localization is one of the most important and basic problems in autonomous driving, upon which its reliability depends. The navigation task becomes simplified if the AV can match the perceived environmental features through its sensors with the *a priori* map of the environment available to it. As such, it can successfully estimate the location of the vehicle [87,88] and detect obstacles based on discrepancies in the *a priori* map and the sensor data. For instance, the LiDAR used for localization and object detection heavily relies on particle filters [89]. LiDAR generates point clouds that provide a shape description of the environment. Thereafter, the particle filter helps to compare the observed shape against a known map to enhance the accuracy of the collected data. A map is composed of landmarks with defined locations and other properties. Map-building includes identifying the unobserved landmarks and classifying and integrating them into the actual map. The features that cannot be identified by comparing them to a known feature are used to create new features within the map maintained by the navigation algorithm. Two types of maps can be defined [90]:
  - Absolute maps describe a place based on its fixed point on a common, global coordinate frame. Landmarks are stationary points in two dimensions that are defined by two parameters indicating their position in the Cartesian plane in relation to some global coordinate frame.
  - Relative maps indicate the relationships between landmarks. The relative map state between landmarks $L_{i}$ and $L_{j}$ is obtained by the vector subtraction of the absolute map locations that are represented as vectors of coordinate values.
  The map-based approach to navigation is only useful in largely unchanging environments having an accurate *a priori* map. However, general navigation means being able to operate in any environment while detecting landmark features through sensors and building a map. The simultaneous localization and mapping (SLAM) approach (depicted in Figure 6) helps the AV determine its location in an unknown terrain with no *a priori* map available. While in motion, the AV relies on its sensors to collect the necessary data about the surroundings and utilizes them to synchronously build a complete map of the landmarks and then, by tracking the relative position between the vehicle and identifiable features, both the position of the vehicle and those of the features can be accurately estimated [91]. In the extended Kalman filter (EKF), the perceived information is continuously fused in a recursive way to receive bounded estimates of the vehicle and landmark features [92,93].
- Decision making: this includes path planning, maneuvering through the traffic and past obstacles, automated parking, and following vehicles, among others, without human driver interposition. The main challenge faced by drivers on the road is to cope with the possible actions of other vehicles in their vicinity. The decision unit of an AV solves the aforementioned problem by accurately predicting the actions of other vehicles using a stochastic model of some of their predicted position sets and associates these sets with a probability distribution [94]. Thereafter, the decision on the next action plan is taken based on how well those probabilities align. To enhance the efficiency of the decision making process, AVs are equipped with AI-capable systems such as speech recognition, steering control, eye tracking, economical fuel, gesture control, etc.
  The path planning of an autonomous vehicle in a dynamic environment is one of the most challenging aspects. Conventional path-planning methods include some time-consuming and computationally intensive approaches such as SLAM, distance control algorithm, bumper field approach, lane curvature, vector field histogram, stereo block matching, etc. [95]. Therefore, to bypass this computational complexity, probabilistic planners are used [96]. Besides that, AI-based approaches such as neural networks, fuzzy logic, simulated annealing (SA), and genetic algorithms are used, which lead to high accuracy. In [97], authors proposed using neural networks (NNs) to perceive the surroundings and adaptive finite state machine to help the AV navigate its way through urban environments. Similarly, conventional linear car-following models are proposed to be replaced by AI-based ones, using algorithms such as CNN, reinforcement learning (RL), and inverse RL (IRL). In addition to these, ML and/or DL approaches can also utilize the images, vehicle speed, and steering angle as input and control the steering angle and speed to follow cars. For instance, the fuzzy logic and genetic algorithms were combined to control the lateral information of steering wheels [98].

### 6.2. Edge Computing Platform for Autonomous Vehicles

Cloud computing is not an effective solution to handle the IoT platform needed in autonomous vehicles due to the following challenges [50]:

- While on road, an enormous amount of real-time data (approximately 2GB/s) needs to be processed by AVs [99] in addition to making robust decisions on how to steer the car with latency constraints. The AV cannot afford to transfer such a huge amount of data to the cloud instantaneously and wait for its response on how to tackle a situation. The high latency incurred in such a case can jeopardize the AV as well as the passengers on the road and can even be fatal.
- The transfer of such an enormous amount of data over the cloud network also leads to overheads that reduce throughput and increase energy consumption, network traffic, and cost.
- The cloud data center, where the data are offloaded, may be located in a faraway geographical region, which may add to the latency incurred in the network since it increases with the physical distance between the cloud and the device.
- The data generated by the myriads of IoT sensors and devices in the AVs are heterogeneous in nature, which increases the complexity of the cloud.
- Processing large amounts of real-time data sent by a plethora of AVs increases the workload on the cloud.

All the aforementioned reasons have spurred the need for edge computing for autonomous vehicles. In such an environment, AVs will be connected to edge devices (end devices such as IoT devices, mobile phones, and embedded devices that communicate with the edge servers) using wireless communication networks to access real-time data analytics. Communication among vehicles can be realized through dedicated short-range communications (DSRCs) or device-to-device communications [100]. Since AVs do not run computationally intensive applications all the time, a low-latency edge computing system can be used by efficiently managing vehicular resources. Despite several studies on cloud computing systems for vehicular environments, various limitations pertaining to infrastructure requirements being usable in static scenarios or being application-specific have remained. To overcome the deficiency, Feng et al. [101] proposed an autonomous vehicular edge (AVE) framework, wherein they introduced workflow to allow the autonomous organization of a vehicular cloud.

**Figure 7:**
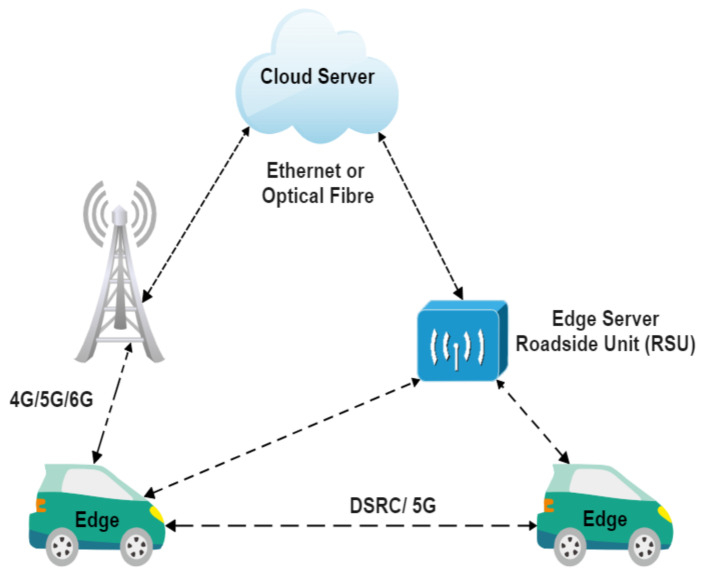
Autonomous driving edge computing system [12].

V2X is emerging as one of the popular ways to reduce the cost associated with huge computational demand on autonomous driving edge computing systems as depicted in Figure 7. There is an extensive research focus on V2V and V2I since it encourages information sharing among vehicles and computation offloading to the road side units (RSUs). In V2V, even if two vehicles are not connected wirelessly, other vehicles would transfer the message between them.

Cooperative autonomous driving [102] is one significant offshoot that leverages V2X communications to enhance the efficiency of the vehicular edge system. It has two categories [12]: (1) Cooperative sensing that shares information among AVs through V2V and V2I communication. It increases the sensing range of an AV and makes the system more robust. The reliance on roadside sensing infrastructure will also reduce the need and the cost of many expensive onboard sensors, making this approach highly cost-effective. The second category is (2) cooperative decision in which a group of autonomous vehicles cooperate among themselves and make decisions based on the information shared among each other.

### 6.3. Edge Intelligence: Levels, Architecture, and Technologies for Distributed Training

**Figure 8:**
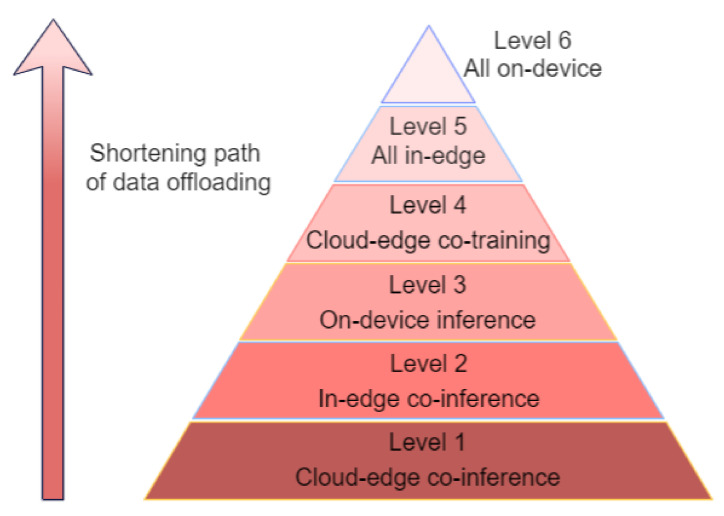
The six levels of edge intelligence [103].

Edge intelligence should be able to utilize the data and resources distributed across end devices, edge nodes, and cloud data centers to fully unleash the potential of the DNN model by improving its training and inference abilities. This does not imply that all the training and inference of the model will be carried out on the edge but it should be able to work in cloud–device–edge coordination via data offloading [103]. Edge intelligence can be divided into six levels according to the amount and path length of data offloading. The six levels are illustrated in Figure 8. Ascending to higher levels reduces the path length of data offloading. Hence, the transmission latency of the offloading process to the server decreases, privacy improves and the WAN bandwidth cost reduces. Unfortunately, this also leads to increased computational latency and higher energy consumption. The six levels are:

- Level 1: cloud–edge co-inference and cloud training constitute level 1 of EI. The model is trained in the cloud but the inference utilizes cloud–edge cooperation. The totality of the data are not offloaded to either the cloud or the edge. Different parts of the data are offloaded to both of them, based on the requirement, latency constraints, and size.
- Level 2: In-edge co-inference and cloud training. The model is trained in the cloud but the inferencing is performed at the edge. In other words, the inferencing is performed within the edge network by completely or partially offloading the data to the edge nodes or edge devices.
- Level 3: On-device inference and cloud training. Training of the DNN model is carried out in the cloud, but the inferencing is entirely performed in a local end device. This implies that no data will be offloaded anywhere else.
- Level 4: Cloud–edge co-training and inference. At this level, both the training of the model and inferencing is performed in a coordinated edge–cloud fashion. It implies that parts of the data are trained in the cloud whereas the tasks on the remaining parts are performed at the edge network.
- Level 5: All in-edge. Here, both the training and inference are performed in the edge server only, without the involvement of the cloud. Here, data are offloaded to the edge nodes.
- Level 6: All on-device. At this level, both the training and the inference are carried out on the local device.

As the data generated from IoT and mobile devices increase exponentially, the task of training AI models becomes more cumbersome. The architectures for distributed training of DNN models at the network edge can be:

- Centralized: The cloud data center will perform the DNN model training using the data gathered from distributed end devices such as mobile phones, sensors, and cars. This is reflected in the edge intelligence levels 1, 2, and 3.
- Decentralized: corresponding to level 5 of edge intelligence, decentralized training does not require the intervention of the cloud data center. Individual computing nodes train their own DNN models using local data, thereby preserving privacy. They can also communicate with each other to provide updates about the local training of the model.
- Hybrid: as the name suggests, it combines centralized and decentralized architecture. The training of the DNN models may be performed in a decentralized way by the interacting edge servers. Alternatively, the centralized way of training can be adopted by the cloud data center. As such, it encompasses level 4 and level 5 in edge intelligence. It is also referred to as cloud–edge–device training given the involvement of all three in the training phase.

To tackle and improve various performance indicators such as the training loss, latency, privacy, communication cost, and others, some key technologies are used to train the AI models for edge intelligence. Most of these training models are distributed frameworks. For edge nodes with limited storage capacity and computing resources, training a comprehensive model on their own is cumbersome. Therefore, distributed training is the way to move forward that encourages coordination between edge nodes. Data splitting and model splitting are two divisions of the distributed frameworks [104]. Model splitting separates and deploys different layers of a neural network on devices by relying on sophisticated pipelines. Moreover, data splitting can be subdivided into master–device splitting, helper–device splitting, and device–device splitting. The key differences among these subdivisions lie in the place of procurement of the training samples and how the final global model is assembled.

Besides distributed frameworks, knowledge distillation-based frameworks are mostly non-distributed as they rely on transfer learning techniques [105]. In it, firstly a simple network is trained on a basic dataset. Then, the learned features are transferred to different student networks to run on respective datasets.

Some of the distributed model training frameworks are discussed briefly below.

**Figure 9:**
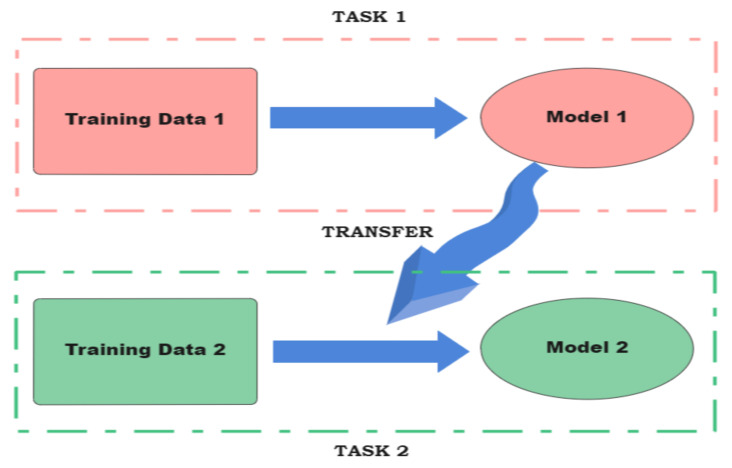
Knowledge transfer learning.

1. Federated learning: this aims to preserve privacy while training the DNN models in a distributed manner. Instead of transmitting the data generated by multiple clients to a centralized server for training, it leaves the raw distributed data with the clients. It trains a shared model on the server by accumulating the updates that were computed locally on edge devices [106]. To deal with the optimization problem, the distributed trained gradient updates of each client are averaged to optimize the global model. To enable smoother communication among the edge nodes, the global gradient from the various distributed local models running on them should be optimized. To tackle this issue, distributed edge nodes use stochastic gradient descent (SGD), a simple but widely used gradient descent method, to update their local gradients based on mini-batches or subsets of the entire dataset. The updated gradients are sent to a central node for a global model upgrade. However, if all the edge nodes send their gradients simultaneously, the network may be congested. The authors in [107] proposed a selective stochastic gradient descent (SSGD) that allows selectively choosing to share gradients of the dataset with key improvements to the central node.
  To improve the communication efficiency (lowered by unpredictable networks), [106] proposed increasing the computation of local updates on clients to reduce the number of rounds of sending training updates to the central aggregator. To account for those clients who are under severe computation resource constraints and will be hampered by this proposal, the authors of [108] proposed to reduce the communication costs with structured updates and sketched updates.
2. Gradient compression: Decentralized training faces the problem of communication overhead. To reduce that, gradient compression is an intuitive solution that compresses the frequent gradient information updates sent to the central aggregator. Gradient quantization and gradient sparsification are two ways to achieve this. Gradient quantization solves this problem by quantifying the elements of gradient vectors to a low-bit but finite precision value. On the other hand, gradient sparsification helps reduce the communication overhead by transmitting part of the gradient vectors.
  Gradient compression can be a powerful tool because it has been shown in [109] that 99.9% of the gradient exchanges in distributed SGD are redundant. Therefore, a deep gradient compression (DGC) scheme is proposed that compresses the gradients by almost 270 to 600 times for different types of CNNs and RNNs. Taking cues from this, [110] proposed edge stochastic gradient descent (eSGD). eSGD improves the first-order gradient-based optimization of stochastic objective functions in edge computing. To do so, it does two things: (1) determines and transmits only the important gradient coordinates and (2) tracks obsolete gradient coordinates using a momentum residual accumulation to avoid sparse updates that lead to low-convergence rates. The concise convergence analysis of sparsified SGD was performed using k-sparsification or compression in [111].
3. DNN splitting: this upholds privacy by transmitting partially processed data instead of the raw data. A DNN model can be split between two successive layers. This does not lead to any loss in accuracy. Splitting is conducted between the end devices and the edge server. However, the main bottleneck lies in selecting the splitting point in a way to fulfill the latency requirement of the model. Considering this, the authors in [112] proposed splitting the DNN model after the first convolutional layer to minimize the burden on mobile devices.
  Besides preserving privacy, DNN splitting also helps manage the huge computation burden of DNN model training by introducing parallelization. Two kinds of parallelism are involved in DNN training in parallel: data parallelism (which increases the overhead of communication) and model parallelism. To eradicate the problems associated with them, [113] designed PipeDream, a system based on “pipeline parallelism" that automatically decides how to split the given model across the available computation nodes. Pipeline parallelism frequently injects mini-batches (subsets of the dataset) into the system to ensure the maximum utilization of the computation resources. It is, therefore, an improvement over model parallelism, that may sometimes lead to the under-utilization of the available resources.
4. Knowledge transfer learning: To conserve the energy and computation cost over edge devices during model training, knowledge transfer learning first trains a base (or teacher) network with an initial or base dataset. The learned features are then transferred to a student network to be trained on their respective target datasets (Figure 9).
  Closely related to DNN splitting and inspiring the designs of many frameworks, transfer learning treats the shallow layers of a pre-trained DNN model as a generic feature extractor that is applicable to other target datasets. As a promising solution, it greatly reduces resource demand through learning on edge devices. To provide a thorough investigation into its effectiveness, accuracy, and speed of convergence, the authors in [114,115] conducted extensive studies. They examined various types of student network architectures and forayed into the different techniques that can be adopted while transferring learned features from a teacher network to its corresponding, shallower student networks. As per their findings, transferring learned features from both the intermediate and last layers of the teacher network gives a great boost in terms of performance.

### 6.4. Edge Intelligence Model Inference: Architecture and Technologies

For the high-quality deployment of edge intelligence services, the implementation of model inferencing is critical. Therefore, in this section, we will deal with the architectures and enabling technologies of the DNN model inference at the edge.

In [103], several edge-centric model inference architectures have been described and classified, which are summarized in the follows.

- Edge-based: if the DNN model inference is carried out on the edge, the prediction results will also be transmitted back to the edge server, from which the device had received the input data. This approach will make its implementation easier on various mobile devices since inferencing the model on the edge can enhance the proximity between the edge server and edge devices, as compared to when it is performed on the cloud.
- Device-based: the device receives input data from the edge and does model inferencing locally. It requires considerable computation resources such as CPU, GPU, and RAM on the mobile device. The security and reliability of this approach can be ensured because no communication is established with the edge server while the process continues. However, the performance entirely depends on the capacity and efficiency of the mobile device itself.
- Edge–device: the first step in this is DNN splitting, based on the network bandwidth, resources available, and current device workload. The device will execute the model until the splitting point layer, with the intermediate data transmitted to the edge. The edge will process the remaining layers and send the prediction results back to the device. In comparison with the aforementioned architectures, this offers more flexibility. The only drawback is that it requires higher computation resources in the mobile device executing the convolution layers at the beginning of the model, which is usually computationally expensive.
- Edge–cloud: It is executed in a way similar to edge–device architecture. The input data are obtained at the mobile device and the DNN model is then executed in a coordinated manner between the edge and the cloud. It is beneficial for devices facing high resource constraints.

**Figure 10:**
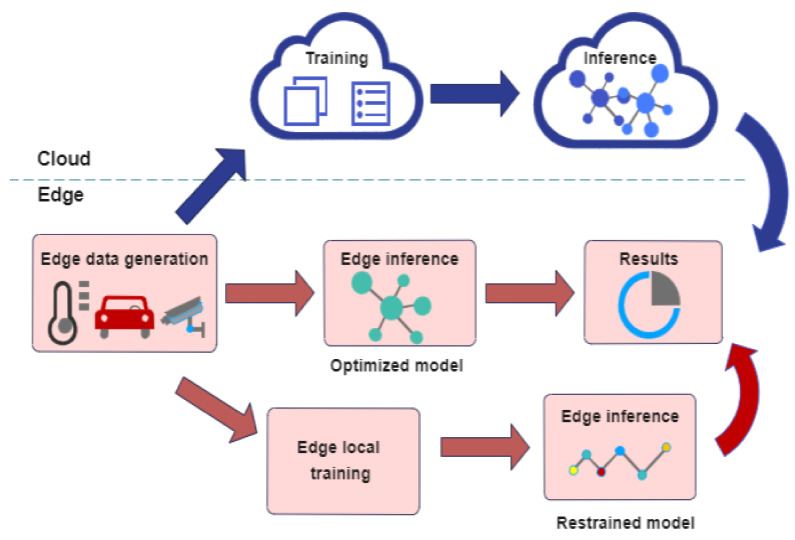
Flow of data in an edge intelligence architecture.

Figure 10 illustrates the data flow in an edge intelligence infrastructure, showing the training and model inference steps in both cloud and the edge.

Some of the enabling technologies for model inference are discussed briefly below [116].

1. Model compression: this tackles the compromise between resource-desiring DNN models and resource-constrained edge devices. It reduces model complexity and resource requirement and enables model inference to be carried out on edge devices, which in turn will reduce system latency and strengthen privacy. Some of the technologies to compress and quantize weights include singular value decomposition (SVD), Huffman coding and principal component analysis [116]. Among the various methods adopted for model compression, weight pruning is the most widely adopted technique. It first ranks the neurons in a trained DNN model based on their importance and contributions. Thereafter, it removes the lower-ranking weights (or neurons) to reduce the model size. However, the reduction in the accuracy of the network with the removal of neurons is a key challenge, which was tackled in a pilot study in 2015 [117]. In their magnitude-based weight pruning method, they removed the smaller weights with magnitudes under a threshold value (e.g., those under 0.01 or 0.001) and then fine-tuned the model to restore it to its old efficiency. However, for energy-constrained edge devices, this may not be beneficial because it was observed that the reduction in small weights does not significantly contribute to the reduction in energy [118].
  Another noteworthy technique is data quantization. It adopts a more compact format than the 32-bit floating point format to represent weights and layer inputs. It compresses the data to represent these by numbers with lesser bits. Hence, it reduces the memory footprint, accelerates computation, and improves energy efficiency. However, to meet the diverse requirements of latency, energy, resources, and computation power, two or more techniques are used in tandem. For instance, both deep compression [119] and Minerva [120] use weight pruning together with data quantization to lower the latency, reduce power consumption, and enhance the accuracy of DNN model inference.
2. Model splitting: to reduce the computation pressure on resource-constrained end devices, model partition/splitting is an intuitive approach that offloads the computation-intensive part of the model to the edge servers or nearby mobile devices. This leads to better performance in terms of latency, energy, and privacy. Model partition falls in two types: (1) partition between edge server and device and (2) partition between devices. As an example of the former type, Neurosurgeon is an iconic effort [121]. The authors proposed a regression-based method to determine a suitable partition point in the DNN model after analyzing the latency of each layer. This leads to optimal model inference performance in terms of latency and energy requirements. Another example is [122], wherein the DNN model is split into two parts and run collaboratively on the edge and end device. The computation-intensive part is taken care of by the edge server whereas the remaining part is performed on the mobile device. In such an approach, the main challenge lies in accurately identifying the point of splitting of the model layers and when to exit it, depending on the inference accuracy constraints.
  As an instance of the latter type, MoDNN [123] is one such pioneering work, in which they built a local, micro-scale distributed computing cluster in wireless local area network (WLAN). It can be used to split a previously trained DNN model across various mobile devices with licensed Wi-Fi connections. The mobile device carrying out the DNN task is the group owner, while the rest of them act as worker nodes. With an increasing number of worker nodes, the DNN computation process becomes accelerated in a similar ratio.
3. Model early exit: To accelerate the model inference process, model early exit leverages the output result obtained from the early layers of the DNN model to obtain the classification data. Therefore, only a part of the DNN model is utilized for inference. Since a highly accurate DNN model comprises a lot of deep layers that consume a lot of resources during computation at the end devices, tackling latency is the optimization target of this method. A popular adaptation of the model early exit method is BranchyNet [124]. In the original, standard DNN model, several exit branches are added among intermediate and final layers. These exit branches are like exit points that share a part of the DNN with the standard DNN model. Several frameworks have been built on BranchyNet, such as DDNN [125] (distributed deep neural network) across cloud, edge, and device, and Edgent [122,126], which surveys the accuracy–latency tradeoff while simultaneously implementing the model early exit and model partition.
  Besides BranchyNet, other frameworks such as Cascading network [127] add an additional max pooling layer and a fully connected layer to the standard DNN model to accelerate the process by nearly 20%. DeepIns [128] uses model early-exit to propose a manufacturing inspection system for the smart industry. With edge devices collecting data, the edge and the cloud servers act as the first and the second exit points, respectively.
4. Model selection: The central theme on which model selection revolves is that we can adequately select a previously trained DNN model for online inference. It is similar to the model early exit because the exit point of the early exit mechanism can be perceived as a DNN model. However, the key difference lies in the fact that in early exit, the exit point shares part of the DNN layers with the main branch, but in model selection, the models work independently. In [129], a small yet fast model was trained to classify the input data and was used instead of the bigger model when its accuracy exceeded a pre-specified threshold value. In [130], the authors proposed the design of a framework that selects the best DNN models for latency and accuracy, using a trained model selector for various input images. The authors in [131] reshaped the adaptive DNN model selection issue as an optimization problem of hyperparameters by considering the latency and communication constraints. The problem is solved by Bayesian optimization (BO) which improved the minimum energy consumed per image.
5. Input filtering: It is another adopted framework to accelerate the DNN model inference, pertaining to video analytics. It removes those frames containing redundant, non-target objects of the input video data to reduce redundancy in the computation of model inference and the avert loss of computation resources and energy. This also improves the latency and the model inference accuracy. NoScope [132] proposed to accelerate video analysis by implementing a difference detector that will search for temporal differences across frames. It will skip the frames with little to no change in the objects and the rest will be processed by the DNN model inference. Lightweight binary classifiers are used in the difference detector.
  The aforementioned example describes model inferencing using a single camera, which is much less challenging than cross-camera analytics applications that have to detect associations across frames and across multiple cameras. When it comes to DNN model inference using cross-camera (multiple cameras) analysis, ReXCam [133] anchors on a learned spatiotemporal model to filter those redundant frames which are not spatially or temporally associated with the current target’s identity. Since the cost and latency increase with the number of cameras, ReXCam reduces the computation and data burden on the devices by collaboratively using multiple cameras and filtering out redundant frames, thereby improving the DNN model inference accuracy by 27%.

### 6.5. Challenges

Several challenges are identified below:

- Sensor issues: the accuracy of an AI-based model heavily depends on the data obtained by the many sensors available, such as cameras, LiDAR, radar, SONAR, GPS, among others. Sensor fusion is an important aspect of an AV, since fusing data from various sensors accumulates their advantages and leads to better overall accuracy. For instance, the fusing of LIDAR and camera data leads to better performance under poor light conditions [134]. Moreover, different layers of sensors with overlapped sensing areas contribute to redundancy, which ensures high reliability. Abnormal weather conditions seriously hinder the performance of the sensors but sensor fusion can improve the condition by some margin. Complex urban areas and unknown terrains pose a different type of problem in perception that can be improved using SLAM and similar algorithms. The trade-off between the cost of sensors and their accuracy leads to a difficult compromise for AV manufacturers, which ultimately leads to different stakeholders opting for diverse sensors. Thus, the sensor discrepancy leads to heterogeneous datasets. Even the data quality and reliability of different sensors are important questions to think about and work upon. Moreover, the synchronization of the data obtained from various sensors on board, sensors of other vehicles, and different road-side units (RSUs) is an important question. Handling sensors with varying frequencies and timestamps are still a bottleneck since time synchronization accuracy affects the safety of the vehicle. There lacks a universally defined standard for sensor failure [135] or comprehensive study on sensor failure detection. The situation is hazardous since the all-around safety of the vehicles heavily depends on the multiple sensors present. If any sensor failure goes undetected, catastrophic and fatal accidents are bound to take place. Not just technical failures but even the data supplied by the sensor (which itself is working fine) may be wrong due to multiple external factors such as blockage due to dirt, shadows, or deviation of the sensor due to wind force [136].
- Complexity and uncertainty: The uncertainty associated with the AI approaches can be grouped into two categories: (a) uncertainty induced by datasets (sensors may not work properly under different environments, thus bringing multiple unexpected errors), and (b) uncertainty born of implemented models (depending on the functional requirements of the AI-models with the assumption that the sensor data are reliable enough to meet their needs but the unpredictable and dynamic nature of the environment thwarts that perspective [81]). Even malicious attacks can trigger new uncertainties and complexities since they mostly do not need physical access to the vehicular components. In unfamiliar and challenging scenarios such as abnormal weather conditions, road blockage, or severe occlusion, the state-of-the-art algorithms deployed and executed cannot guarantee correct outputs due to deviation from ideal conditions [137]. For instance, lane detection accuracy is lowered at night due to the difficulty for algorithms to utilize prior information from datasets [138]. However, in such situations, humans can fill in the occluded parts of the context and come out safe. As such, the persistent challenge is to develop advanced algorithms to improve detection accuracy, especially in unpredictable situations.
- Hardware problem: the implementation of AI in AVs heavily depends on computing devices to handle the huge number of demanding tasks [78]. These devices are integrated into the AV architecture itself, such as multi-core CPUs, GPUs, distributed systems, and others. For instance, BMW had manufactured AVs equipped with a standard personal computer (PC) and a real-time embedded personal computer (RTEPC), which were connected to the actuators. The PC fused the sensor data to perceive the surroundings and the RTEPC was responsible for controlling the steering, braking, and control of throttle [139]. Though the aforementioned hardware has been successfully tested in real-time scenarios, the main bottleneck is the trade-off between the field test performance (accuracy, latency, etc.) and the cost. Thus, further research must be conducted to overcome the technical challenges while catering to the market needs for the large-scale manufacturing of AVs. With the advancement of the DL approach, GPUs have gained popularity over CPUs due to their intrinsic parallel structure that achieves better efficiency than CPUs while computing large volumes of data. On the other hand, GPUs consume higher power and lead to more significant heat dissipation and an additional power system load. Therefore, field-programmable gate array (FPGA) comes into the picture, which is an integrated circuit that can be configured/programmed by a user to any digital circuit one wants. They have specific computing architectures to be configured into various types of digital circuits, thereby reducing the engineering costs. FPGA thus outperforms CPUs and GPUs due to higher computing efficiency and lower power consumption. Besides the aforementioned, new and advanced system architectures are being researched and tested to provide an efficient implementation of DL approaches. Despite all that, deep chasms in hardware improvement and implementation in real-time scenarios are present in the large-scale commercial applications of AVs.
- Standardization of safety issues: machine learning algorithms are quite unstable and delicate to use. Minor changes in sensor data, such as cropped images or changes in environmental conditions may put the advanced driver assistance system (ADAS) on the brink of failure regarding detection and segmentation [140,141,142]. Moreover, the ISO 26262 standard [143] for automotive safety was published without considering deep learning, so it lacks adequate ways to incorporate safety issues when linking AI with AVs [144].
- Model training: to ensure proper implementation of machine learning models in AV, the algorithms have to be trained on representative datasets under all application scenarios. If trained properly, ML models can process large volumes of data and detect anomalies, and test correlations while searching for patterns across the data feed. While running, an AV will face a plethora of situations due to the dynamic nature of its surrounding environment, which need to be covered during model training. Otherwise, the significant functionalities of an AV, such as object detection, perception, SLAM, and decision making will be hampered. The scenarios that an autonomous vehicle may encounter on its journey are mostly unpredictable and numerous, thus bringing challenges in training time-sensitive models on data on the scale of petabytes in size. To tackle the challenge, collaborative training [145], model compression technologies [146,147,148], and lightweight ML algorithms [149,150,151] have been proposed in recent years. It is also important to obtain the accurate coordinates of pedestrians, vehicles, lanes, and obstacles for model training using supervised learning approaches, which is a cumbersome process [152].
- Edge computing system design: one of the foremost and most systematic hurdles faced by autonomous driving systems is the design of an efficient architecture that will deliver greater computing power with reasonable energy consumption even at high speeds. To improve the design and workload of current edge computing systems, an efficient benchmark suite is indispensable to represent the workloads typically used in target applications. These are divided into two categories: datasets and workloads. KITTI [153,154], PASCAL3D [155] for 3D object detection, and the MOTChallenge benchmark [156] for multi-target tracking are some customized benchmarks datasets for each algorithm. Currently, CAVBench [157] serves as a good benchmark to evaluate autonomous driving computation system performance, choosing six parameters as evaluation workloads. However, AVs are evolving faster, which requires further research to include dynamic workloads and data to evaluate emerging autonomous driving usage scenarios. Utilizing heterogeneous computing architectures such as CPU, GPU, FPGA, DSP, and ASIC-accelerated systems can help lower the latency of the algorithms used for localization, object detection, and object tracking, which are computational bottlenecks for an autonomous driving system. Although accelerators such as GPU deliver results at low latency and greater efficiency, their high power consumption and cooling load may confine the driving range and degrade the fuel efficiency of the vehicle [158]. The goal is to deliver high power with a small chip area. However, with a limited chip area, it is difficult to merge various tasks in one accelerator. Nowadays, many studies focus on reconfigurable fabrics. However, the efficient utilization of reconfigurable fabrics in the design of edge computing systems remains a major challenge.
  The runtime layer, in simple terms, connects autonomous driving software and hardware. With increasing heterogeneity, the design of the runtime layer becomes more challenging as it needs to efficiently dispatch incoming workloads. It should also be aware of the new edge clouds available to be able to dynamically dispatch the workloads to them. A middleware layer enables the complicated communications between various services present in robotic systems, such as the autonomous vehicle. However, current middleware lack the reliability and robustness to ensure the full safety of an autonomous vehicle. The scalability of the middleware layer should improve with minimal computing overhead and memory footprint. The complex interactions with various autonomous driving edge computing architectures should be seamless enough to enable smooth client and cloud communication.
- Infeasible testing: machine learning algorithms used in AVs are trained using a large number of datasets. The model is then stored in a set of weighted feature combinations, which can be difficult to test thoroughly [159]. Currently, the environment in which they are tested is of three types: simulation, experiments with model vehicles, and experiments in the real world. The first two are widely used across most research, whereas testing in the real-world is not yet widely conducted. This leaves serious gaps in the accurate training of the AI algorithms implemented because nothing can simulate the performance of the vehicle in real-world circumstances riddled with uncertainties and challenges. In a previous study, [160], it was estimated that that approximately $10^{9}$ hours of vehicle operation would be required to verify the catastrophic failure rate and the test should be repeated a number of times to obtain data of statistical significance [144]. However, there have been notable instances of successful testing in real-world setups that have highlighted the limitations of existing techniques [161,162,163]. Such real-world experiments should become more widespread in the upcoming years and their collected data should be shared and mined to advance learning and push the autonomous vehicle sector to its final market-ready status.
- Content placement at the wireless edge: The contents specific to automated driving systems have large data volumes. However, the cache space at base stations is limited. Efficiently managing the caching of service contents will improve the cache hit ratio, a higher value of which indicates a reduction in duplicate transmissions and improvement in latency [164]. Caching some of the most popular contents at the base stations had emerged as a promising solution [165]. The popularity of content is determined by the number of requests for that content as a proportion of the total number of requests. However, the bottleneck that has been overlooked is that content popularity varies with time depending on the dynamic traffic conditions. Current content popularity cannot predict popularity in the near future. Therefore, the onus falls on the edge server to accurately predict the short-term popularity of the content.
  The dynamic location dependency of autonomous vehicles introduces severe spatial constraints on content dissemination. It is not feasible for the edge server to reduce the size of the data chunks transmitted through the cellular networks to share with other vehicles while satisfying the content delivery deadlines. The fast-changing topology of vehicular networks and the spatial distribution of data chunks determine how quickly the content can be delivered to the required systems [166].

## 7. 5G Communications

Over the last two decades, there has been precipitous development in wireless communication technology. We are well aware of first, second, third, and fourth generation mobile technologies, in which the speed and efficiency of wireless mobile networks have improved. In the current scenario, fifth- and sixth-generation networks have attempted to bridge the gaps left by the previous generation of wireless mobile networks, with commendable improvements in latency, speed, system spectral efficiency, throughput per connection, and reliability. As intelligent transportation systems (ITSs) in general and autonomous vehicles in particular gain further popularity with each passing year, vehicle-to-everything (V2X) communications have become an integral part of it. V2X allows wireless connectivity among vehicles, roadside units, pedestrians, passengers, and base stations. Connected AVs (such as an autonomous vehicle platoon) can implement cooperative decision making and cooperative perception using various wireless communication technologies such as DSRC or long-term evolution (LTE). Cooperative planning and perception provide information to vehicles beyond line-of-sight and field-of-view, thereby improving the control over vehicles and their performance [167].

Mainstream vehicular communications have two categories: DSRC- and LTE-based vehicle-to-everything technology (LTE-based V2X or simply, LTE-V). DSRC is bound by the IEEE 802.11p and IEEE 1609 standards for wireless access for vehicular environment (WAVE). DSRC faces bandwidth constraints to deliver a high data rate link and is also prone to malicious attacks. LTE-V is based on the cellular network technologies standardized by 3GPP, a collaborative project among telecommunications associations across the world. 3GPP was initially founded with the goal of developing specifications for third-generation (3G) mobile systems but has now expanded into 5G, 6G, and beyond. The 6 GHz bandwidths used by fourth-generation LTE advanced (4G LTE-A) are highly congested, leaving minimal space for utilization by autonomous vehicles. This limitation can also hinder the application of various AI algorithms which require the collection and dissemination of large volumes of real-time data [82]. This has spurred the fifth-generation (5G) communication to exploit the under-utilized mmWave bands (10–300 GHz). mmWave bands in that range are underutilized because of high penetration loss and path loss. mmWave bands are indispensable for 5G V2X communications because recent studies have shown the necessity of multi-Gbps links to enable such communications [168,169]. Therefore, to provide a solution to the complexity of directional mmWave communication, the authors in [170] proposed an online learning algorithm to solve the beam selection problem in mmWave vehicular systems.

Autonomous or semi-autonomous vehicles connected by 5G technology are predicted to be better supporters of the various AI algorithms required for environment sensing, perception and decision making. It is anticipated that 5G technology will bring a paradigm shift in the capability of mobile networks and their implementation in ITSs, smart healthcare, and unmanned aerial vehicles (UAVs), to name a few.

### 7.1. Implementing 5G Technology in Autonomous Vehicles

Fifth-generation targets for highly scalable, convergent, and ubiquitous connectivity will play a significant role in ushering in new services, applications, and use cases. V2X communication facilitates autonomous driving and is one of the major 5G use cases. It is predicted that V2X will impact and change transportation in the future. As described by 3GPP, vehicle-to-infrastructure (V2I), vehicle-to-network (V2N), vehicle-to-pedestrian (V2P), and vehicle-to-vehicle (V2V) communications constitute the four types of connectivity of V2X. V2X allows the realization of the requirements of autonomous driving, including coordinated driving and path planning, real-time 3D mapping, sensor data sharing, remote driving, and vehicle platooning [171].

**Figure 11:**
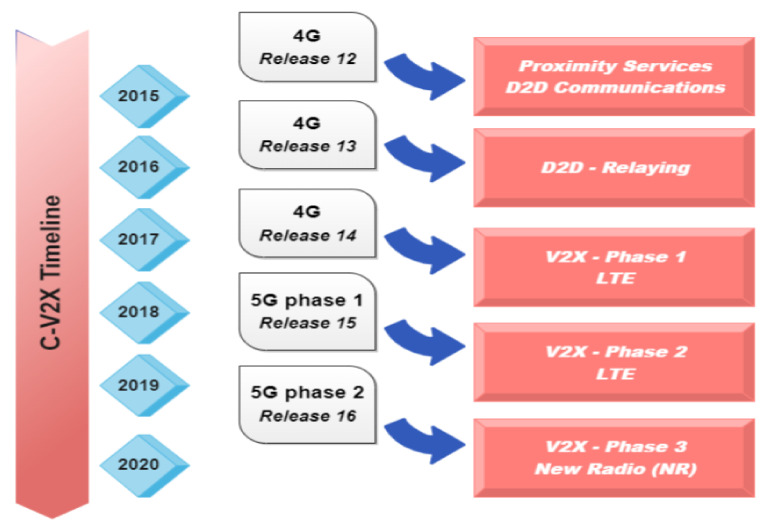
The six levels of edge intelligence.

Two types of V2X operation are supported for autonomous driving in LTE and 5G. One relies on network infrastructure and the other is device-to-device (D2D) communication, also known as sidelink [172]. Starting with 3GPP Release 16, the 5G New Radio (NR) (the brief timeline shown in Figure 11), which is a global standard for a stronger and more capable cellular network, enhances the ultra-reliable low-latency communication (URLLC) and offers higher throughput.

URLLC is the most promising addition to the myriad capabilities of a 5G network. It is a game-changer for latency-sensitive devices and operations such as factory automation, autonomous driving, industrial Internet, smart grid, and robotic surgery. URLLC will significantly lower the latency, including the time for the random access procedure [173]. Time-sensitive networking, wherein all the devices will be synchronized to the same time-base to have a common viewpoint from the time domain, will help in lowering the latency and allow traffic shapers used for managing traffic to be time-aware. However, URLLC is the hardest to secure due to the need for strict QoS, which is different from that required for mobile broadband services. NR has a number of novel features. The first is flexible sub-carrier spacing, which can be $2^{n}$ multiples of 15 KHz for *n* an integer in the range of 0–5. Known as 5G NR numerology, different sub-carrier spacing can be used to meet different requirements of autonomous driving, such as high mobility and low latency. In [174], it is shown that large numerology helps reduce inter-carrier interference caused by the Doppler effect but makes it more vulnerable to inter-symbol interference due to multipath propagation in a V2X scenario. Another feature is network slicing. Network slicing is the logical division of the physical end-to-end network to satisfy different service requirements. Each slice can be customized to meet the needs in terms of latency, throughput, and other metrics. Slice isolation can be implemented to safeguard individual slices from the influence of other slices, thereby improving the security of NR-V2X. More effective channel coding schemes are adopted in 5G NR. The low-density parity check (LDPC) code is used in the data plane, and polar code is used in the control plane to accelerate the decoding process. Fast decoding is beneficial to autonomous driving as it reduces latency. In order to meet the requirements posed by a wide range of use cases in vehicular networks, NR-V2X supports three types of the cast, which is more than LTE-V2X. The cast types are [175]:

- Unicast: it enables direct communication between a user equipment (UE) pair.
- Groupcast (multicast): a transmitter UE sends messages to a set of receivers that meet certain criteria such as being a member of a group.
- Broadcast: the message sent by a single transmitter UE is received by all UEs within the transmission range of the transmitter UE. Then, they decode the message and perform the necessary tasks such as transmitting it to other UEs in the area.

The different types of data transmission allow different types of information to be sent properly to save bandwidth and enhance privacy. In order to improve the performance of unicast and multicast, a physical sidelink feedback channel has been added to avoid the overhead associated with blind re-transmission. In NR-V2X, the resources needed for D2D communication between vehicles can be dedicated (overlay) or shared with other cellular users (underlay). Two sidelink modes manage the scheduling of resources. Mode 1 assumes the full coverage of vehicles by base stations, whereas Mode 2 does not impose such a requirement. Mode 1 allocates resources in a pre-configured or dynamic manner. In Mode 2, the distributed resource allocation is used and four different sub-modes are defined to allow more flexible resource scheduling for various situations. In [176], a thorough performance study of Mode 2 resource scheduling was conducted, taking into account vehicle density, the physical layer (PHY) and medium access control (MAC) settings, and traffic patterns. The ultra-reliable and low latency features of 5G can be used to the advantage of autonomous vehicles. In [177], 5G, network function virtualization (NFV), and network slicing were employed to reduce end-to-end latency which includes propagation latency, queuing latency, and handling latency. In order to reduce the delay, the core network comprises the application, control, and data planes. The control plane is further divided into cloud, fog, and edge layers. Each layer performs different functions. For instance, the fog control plane is responsible for handover between base stations. Numerical analysis and simulation based on NS-3, vehicles in network simulation (Veins), and simulation of urban mobility (SUMO) show that the proposed architecture is more scalable for autonomous driving traffic compared to best-effort traffic.

### 7.2. Main Constituents of 5G for V2X

**Figure 12:**
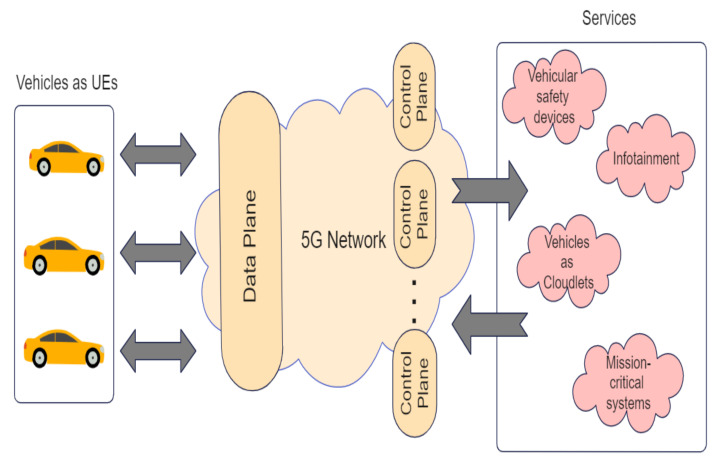
The network slicing architecture for vehicular communications.

Besides supporting new air interfaces and access technologies over the newly assigned spectrum, 5G will leverage the architecture of current wireless technologies such as LTE, carrier aggregation, high-speed packets, etc. This will ensure that 5G is interoperable with those technologies and can deliver the performance and QoS desired by vehicular communications. However, this does not mean it lacks intrinsic constituents vital for the coexistence of diverse technologies on a common, unifying platform. The authors in [178], besides assessing the existing standards of vehicular communications and the most promising aspects of 4G LTE that can be adopted in 5G, presented a study of the crucial building blocks of 5G technology relevant to vehicular communications (V2X). These are described as follows:

- Proximity service: proximity service (ProSe) is a device-to-device technology that was introduced in 3GPP’s Release 12 [179]. This allows the detection and direct communication between nearby UEs in both licensed bands (such as LTE uplink spectrum) and unlicensed bands (such as Wi-Fi, Bluetooth). A number of advantages, such as scalability, manageability, privacy, security, and battery efficiency, distinguish ProSe from existing D2D technologies. Instead of relying on the network for location discovery (for example, Facebook Places), ProSe provides ad hoc location discovery without the requirement of an infrastructure. Fulfilling the service requirements of public safety communications allows first responders to communicate directly when the infrastructure-based communication is unavailable or has failed.
  Considering 3GPP’s introduction of PC5 interface [180], a new direct link called “sidelink" was introduced in the access stratum layer. It can be utilized by the ProSe-enabled UEs to communicate among themselves when they are nearby. The UEs can transmit and receive data without the need for evolved Node B (eNodeB) to discover and synchronize with each other. The UEs can advertise and detect useful information without establishing a communication link. Synchronization allows UEs in proximity to agree on common information and decode sidelink transmissions.
  The various shortcomings of the IEEE 802.11p standard, such as latency issues, wrong transmissions, interference, and channel saturation in networks can be overcome by ProSe. Thus, vehicles can detect others directly or through the locally routed data path using the periodic exchange of beacons [178]. Besides enhancing vehicular safety communications, ProSe can be used to track down security attack sources in AVs, by exploiting the locally routed data path.
- Network slicing: to meet the myriad performance requirements (latency, reliability, availability, scalability) of services, such as the automotive industry, healthcare, and smart manufacturing, the supporting network should be flexible enough to accommodate them besides the existing service requirements. This can be achieved by Network Slicing. As the name suggests, it refers to slicing up the physical network infrastructure into logical segments, called slices. The idea is demonstrated in Figure 12. It helps in the better management of multiple access networks by splitting the single control plane into several control planes for specifying the forwarding rules of the given data plane. The slices can be controlled by individual slice owners, which are of two types: the over-the-top (OTT) service providers and virtual mobile network operators (VMNOs) [181]. The slices are allotted different network functionalities, using technologies such as software-defined networking (SDN) and network functions virtualization (NFV).
  Various network slicing procedures have been discussed in the literature, such as making use of an efficient scheduling algorithm based on a centralized SDN architecture over a flexible cloud radio access network [182], proposing an architecture based on autonomic network slicing with a provisioning and reconfigurable module based on centralized SDN architecture [183]. The authors in [184] and [185] proposed some new schemes to support 5G network slicing for the automotive verticals, out of which one slice was allocated to autonomous vehicles. Their proposed framework, based on a centralized SDN and NFV architecture, comprises a set of network slices representing various use cases of V2X. It depends on V2V communication as the radio access technology. However, the absence of a mathematical framework in [184] and [185] to define the main requirements of autonomous driving (reliability and ultra-low latency) needs to be looked into. Moreover, assigning a single slice to autonomous driving is insufficient to handle the different functionalities of an autonomous vehicle. In contrast, the authors in [177] proposed a hierarchical and decentralized SDN architecture with three layers (fog, edge, and cloud layers), coupled with NFV technology to deliver better QoS to autonomous vehicles. Assigning service slices to the four main functionalities of an autonomous vehicle, namely localization, perception, planning, and system management, will allow efficient access to them and satisfy the low latency requirement.
- Mobile edge computing (MEC): With the exponential increase in mobile applications and mobile data traffic, network operators have to work harder to keep up with the surging demands. In 2013, Nokia introduced the concept of mobile edge computing [186]. As a one-stop solution to the bugging problems, mobile edge computing brings mobile applications closer to the edge, allowing the execution of functionalities in proximity to end users [65]. Therefore, MEC can deliver the strict latency requirements of 5G vehicular communications (100 ms for safety) and ultra-low latency (1 ms for autonomous vehicle use cases). This can meet the standards of high bandwidth efficiency, better QoS, routing area code (RAC) information, and location awareness [187]. To make the services of MEC available to users, they have to be virtualized by NFV. The standards of MEC architecture and the offering of cloud-like facilities at the edge were proposed by ETSI [188]. MEC promised a paradigm shift in the development of different network services and applications. Some of the main use cases of MEC are distributed content delivery, caching, web performance improvement [189], computational offloading [190], etc.
  However, why is MEC so significant when cloud, edge, and fog computing already exist and can be leveraged for use in AV architecture? Many computationally intensive applications such as computer vision, artificial intelligence, etc., need real-time data to work. Traditionally, offloading the data from sensors and actuators to the remote clouds for processing cannot meet the strict latency constraints of these services and leads to heavy backhaul usage. Hence, the distributed architecture of MEC deployed close to the end users can optimize mobile resources by computing and caching computation-intensive services at the network edge, while the rest can be computed at the local or regional clouds [191]. Pre-processing large data before data offloading will reduce the burden on clouds and allow them to be more efficient. Additionally, MEC provides network improvements such as context-aware services utilizing radio access network (RAN) information such as user location and allocated bandwidth.

### 7.3. 6G Communication

Since 5G has adequately matured and entered the commercial operation stage, academia and research organizations have shifted attention to sixth-generation (6G) communications. Even though the necessity for 6G has been questioned [192], many new scenarios are believed to be the driving force behind 6G. Applications that will benefit from 6G include holographic telepresence, immersive extended reality, digital twins, robots, haptic Internet, remote medical surgery, and autonomous vehicles [193]. In addition, the rapid increase in the number of IoT devices calls for novel methods to meet the connection needs of the network. These applications involve the transmission of a huge amount of real-time data and require low latency, high reliability, and security. It has been perceived that these requirements exceed the capabilities of 5G and necessitate the development of 6G. As 6G is still in the early phase of conception, many different visions and technologies have been proposed. A number of research and experimental implementation efforts have arisen globally. It is anticipated that a concrete consensus will be reached by the year 2030.

**Figure 13:**
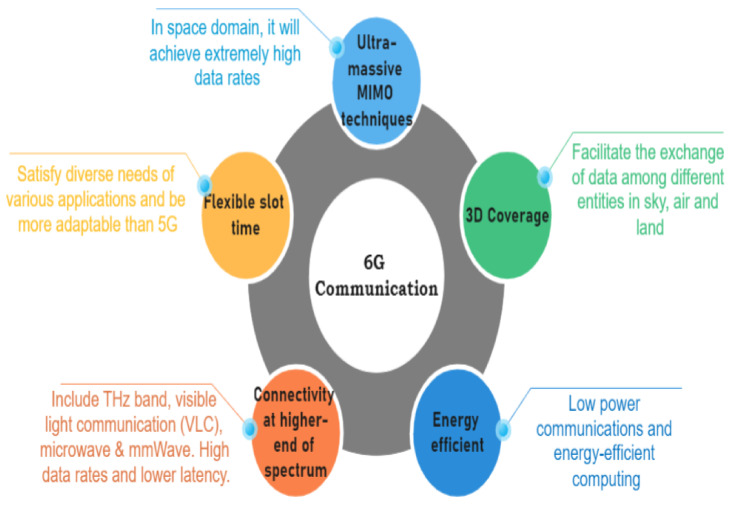
Features of 6G communications.

Conceptually, 6G will exploit the degrees of freedom in space, time, and frequency domains to provide an extremely high data rate to support the demanding application scenarios [194]. In the space domain, ultra-massive MIMO techniques will be used to achieve super-high data rates. Moreover, 3D coverage, including sky, air, and terrestrial, will facilitate the exchange of data among different entities. UAVs [195], low-Earth orbit satellite constellations, and other high-altitude platforms [196] are seen as essential constituents of the 3D structure. In the time domain, the flexible slot time will be in place to satisfy the diverse needs of various applications. The adaptability that has been witnessed and lauded in 5G will be more pronounced in 6G. In the frequency domain, 6G is expected to move to the higher end of the spectrum to reap the higher bandwidths available. These include the THz frequency band and visible light communication (VLC) [193]. Thus, 6G will span across microwave, mmWave, THz, and VLC. These salient features set 6G apart from 5G Figure 13. However, hardware requirements to effectively combine different frequency bands will pose grand challenges to realizing the benefits. In addition, sophisticated channel coding and forward error correction codes need to be developed, especially when the application scenarios make it difficult or impractical to re-transmit packets. High mobility is another technical aspect that needs to be accounted for since high-speed vehicles will be commonplace in the foreseeable future. New handover schemes and cell-free architecture have been proposed to tackle the issue, but further investigation is needed.

As can be seen, a plethora of issues remain to be resolved in 6G. Resource allocation, mobility management, random access, 3D coverage, and coding are just some of the issues, not to mention the intricate interplay among the various degrees of freedom and battery sustainability. The complexity of the issues in 6G is much higher than that in 5G. It has been envisioned that many of the problems are highly complicated and challenging to solve using conventional approaches. AI, machine learning, and game-theoretic methods have been seen as capable of providing solutions to these intractable problems [197]. The discussions in other sections of the paper are relevant. In the context of autonomous vehicles, the advent of 6G will bring lots of advantages in terms of reduced response time, heightened intelligent sensing of the surroundings, better and appropriate reaction, and more agile maneuvering in unexpected situations.

### 7.4. Challenges

The introduction of a number of technical standardization to secure 5G networks has still left some gaps. The identification of the challenging aspects of 5G networks for V2X communications is crucial to find their solutions. This will also ensure the proper implementation of 5G technology in the autonomous vehicle architecture and seamless vehicle-to-everything communication. We hereby discuss the research roadmap of 5G technology for V2X [198]:

- Architecture design: the 5G architecture for vehicular communications should be able to meet all the requirements of an AV. It should be efficient, secure, and compatible enough to support broadcasting data, high data rate, ultra-low latency, and control over a vehicle’s mobility. The scalability of the 5G network is considered to be of the utmost importance in V2X communications. To ensure the smooth transmission of periodic and non-periodic traffic supported by unicast, multicast, or broadcast, the NR sidelink physical layer design requires further enhancements. Besides physical layer enhancements, a number of improvements in the protocol layer are indispensable to meet the reliability standards of advanced V2X communications. One proposed way to enhance reliability is to use a number of blind hybrid automatic repeat request (HARQ) feedback re-transmissions made by the transmitter. However, it will involve high energy wastage due to unnecessary, redundant transmission of the same transport block (TB). Even the obtained efficiency will be insufficient from a system perspective. In [199], the concept of zone overlapping was proposed to enable the HARQ feedback mechanism and resource allocation based on the transmitter–receiver (TX-RX) distance (example, announced in sidelink control information, SCI). For the RX UE to transmit the HARQ feedback, the distance between the zone IDs of TX and RX should be less than the signaled minimum communication range (MCR). Therefore, the size of the zones should be small to reduce the quantization loss in distance calculation. However, smaller-sized zones may not provide enough time for a movable TX UE to sense it before moving on to the next zone, or the remaining useful time may be insufficient. This leads to frequent resource allocation, unnecessary interruption, and degradation in V2X communications.
- 5G transport network: transport network refers to the backhaul of radio base stations or front haul of remote radio units. It is crucial for the improvement and implementation of 5G. The EU project METIS [200,201] defined five scenarios for 5G that will have to be supported in the future. They are: amazingly fast, have great service in a crowd, ubiquitous thing communication (effective support to IoT), real-time and reliable connections, and the best experience follows you. Associating with one or more of these scenarios will introduce various challenges to the network. In 5G, to support very high data rates, a larger number of high-capacity radio access nodes and the densification of radio networks are needed [202]. Thus, the transport network will have to bear high traffic volumes. The “great service on crowd" scenario will prod the transport network to deliver very large capacity data on-demand to particular geographical locations. The “best experience follows you" scenario demands the quick reconfigurability of the transport resources. The other challenges, namely very low latency requirements, low energy consumption, low cost, and supporting a large number of devices, already have competent solutions, which will keep improving as the years go by. Current 5G networks with advanced wireless and optical technologies already produce low latency. However, connecting a large number of devices to the network will generate huge traffic that may lower the QoS of the provisioned services.
- Problems with ProSe: being one of the building blocks of 5G technology for V2X communications, ProSe offers useful advantages. However, there are some bottlenecks that need to be examined and removed for the smooth implementation of the technology. It is of utmost importance for vehicles to discover and communicate with each other while commuting [203]. For vehicles traveling at lower altitudes such as on roads with tall obstacles, the radio interference is higher. For the smooth dissemination of messages, ProSe has to change the base station-to-vehicle communication to device-to-device (D2D) communication. This hierarchical paradigm shift brings with it the challenges of radio propagation.
  Spectrum allocation is another bottleneck for ProSe. In dynamic spectrum allocation, the perspectives of a vehicle can be used as the basis, such as the priority of messages, QoS, and security. In static spectrum allocation, the eNodeB can statically allocate spectrum for vehicles. As part of an IoT environment [204], auxiliary communications among vehicles become important. However, this will exacerbate the interference levels of vehicles using the same band for transmission and reception.
- Network slicing challenges: network slicing, being a relatively new aspect of 5G architecture, has unclear technical specifications and operations, which poses various design challenges for 5G. To give a flexible approach to the sliced networks, it is imperative to identify and classify the myriad vehicular application requirements into technical specifications. Doing this will prevent the slices from affecting each other’s performance and allow them to be seamlessly integrated with one another [178]. Similarly, user requirements should also be categorized, in order to determine whether the network functions should have a centralized infrastructure or should be sliced. Slicing a network can be based on the application QoS requirements, types of vehicular services, and available resources.
- Data management: within the next few years, the amount of data generated by the ever-increasing number of connected devices will increase dramatically. As for vehicular networks, the number of vehicles connected to ad hoc networks under the IoT umbrella has been predicted to rise exponentially. Consequently, processing and storing such gargantuan amounts of data across vehicular networks poses a big challenge [205]. Furthermore, end users expect high data rates along with secure data access. Large distributed networks and huge data generation lead to network congestion while processing data from various geo-distributed database repositories [206]. The high mobility of the connected devices (here, vehicles) and quick changes in topology will also affect data availability and processing at the core data centers. These further give rise to high latency, low bandwidth, high cost, high fault tolerance, and slacked security. That is why real-time data analytics will become more cumbersome in the coming years. Thus, companies need to come up with better data management strategies to deal with such huge volumes of data.
- Mobility management: in the case of vehicle-to-infrastructure (V2I) communications, the main bottleneck is the efficient and secure mobility management as a result of frequent handovers and large-scale vehicular machine-to-machine (M2M) communications. The integration of IPv6 with the existing general security services standards of vehicular cooperative systems as defined by ETSI for intelligent transportation systems (ITSs) has several gaps. In [207], the authors used a vehicular communication architecture based on ETSI/ISO regulations, along with Internet Protocol Version Security (IPVsec) and Internet Key Exchange Version 2 (IKEv2) to secure the IPv6 Network Mobility (NEMO). They analyzed the performance of the secured NEMO on the basis of bandwidth, traffic type, signal quality, and movement speed. 3GPP did not introduced standard optimizations to handle the transfer between LTE and IEEE 802.11p standard and reduce the overhead of security signaling. This challenge becomes more profound when the vehicles are moving fast across different domain operators. Cross-domain handover authentication should be improved and optimized to allow better resource utilization and QoS [208].
- Security: 5G-enabled V2V communication involves cooperative perception and driving, which is a great way to reduce fuel consumption and avert risks associated with driving. In cooperative driving, autonomous vehicles will drive in platoon formation, all the while communicating with each other through 5G or 6G. However, vehicular communications are vulnerable to a variety of attacks that aim to derail vehicles from their path and jeopardize the safety of the passengers. Trusting the data received in V2V and V2I communication can be challenging. An attacker may deliberately send out incorrect messages to divert the path of the vehicle and lead to a fatal crash. One well-known attack is known as the Sybil attack, whereby attackers create multiple false identities to send incorrect information and ultimately gain control of the vehicle [209]. Enhancing ways and devising algorithms to detect and tackle such attacks is highly important. In [210], the authors proposed to help the host vehicle to determine the authentication of the messages received from a target vehicle. The trajectory of the target vehicle is recreated and further points are predicted using V2V messages and an unscented Kalman filter. It is then periodically corrected using 5G V2V multi-array beamforming localization. The estimated position of the target vehicle and that received from V2V are tallied for the discrepancy to classify as an abnormal one. The reference [211] describes several attacks, namely falsification, covert falsification, emergency braking obstruction, and vehicle position hijacking to falsify the leader and members.
  High-precision maps are an integral part of autonomous driving. In 5G V2N services, receiving real-time map updates is critical. However, potential attacks lurking in the received updates are [212]:
  1. Forged identification: the sender of the update, that is, the vehicle, can pretend to be another contrived vehicle and send multiple dubious messages in a short time. The map service provider should check the authenticity of the sender and the updates received from it.
  2. Forged location: the sender may provide a false location update to the vehicle (pretend to be in some other place). Such updates are dangerous as they cannot be traced back to detect the false source. Even with secure and safe hardware, spoofing the GPS signal is not difficult for hackers.
  3. Forged event: the update may include a false event that has not yet taken place, such as a forged accident on a highway. This may confuse and force the receiver vehicle to change its path from a known trajectory to an unknown terrain to avoid facing the false situation made up by the attacker vehicle. Sometimes, the false event may be a result of incorrect sensing by the sensors due to several environmental factors, which are non-malicious in their intent. Therefore, map service providers should check the reality of such incidents by tallying the messages received from other vehicles (a major event or obstacle will prompt a lot of warning messages from other connected vehicles in the platoon).
  Cyberattacks will pose the biggest threats to 5G-enabled vehicles. Hackers can easily intrude and remotely control the vehicle’s parts, including the hardware. Some SDN-specific attacks are denial of service (DoS), distributed DoS (DDoS), man in the middle (MITM) attacks, unauthorized access, and privilege escalation [213].
- Privacy: ensuring privacy in 5G vehicular communications is a significant bottleneck that demands separate attention. Privacy means that users’ sensitive information, such as their location, service preferences, user profile, etc., is preserved and can be only accessed and controlled by authorized users. Since V2X communications form the backbone of 5G-enabled vehicular communications in a platoon of vehicles, the leaking of sensitive information has a high probability. Vehicles resort to cooperative driving due to the wireless network constraints and they communicate through beacon messages that broadcast periodically [214]. Each of those messages contains information about the identity of the vehicle, its dynamic status (location, speed, etc.), and a timestamp. However, such messages are prone to privacy attacks because the sensitive information about the identity and location of the vehicle and consequently, those of the passengers, can be intercepted by malicious entities and misused or tracked. Adopting user credentials such as the username and password can be a way to prove a vehicle’s authenticity to the service providers [208]. However, with a swift increase in the available services and the need for stronger security management, users have to remember different combinations with strict rules. To alleviate the complexity of such a scenario for the users and the service providers, ref. [215] proposes an identity federation solution, which is a single sign-on process. It simplifies the registration and login processes, reduces cost, and efficiently tackles the identity management systems of the service providers.
  The authors of [216] provided an extensive and exhaustive survey of the various threats to the 5G network and categorized the authentication and privacy schemes. However, they left out some issues such as trust and access control. The authors in [217] extensively investigated the present security and privacy challenges and listed the current state-of-the-art issues in 5G wireless networks. They have delved into diverse aspects, including integrity, confidentiality and non-repudiation, authentication and access control, key management, privacy and identity management, trust, intrusion detection, and policy enforcement.

## 8. Blockchain

Blockchain is a peer-to-peer network system that uses technologies such as cryptography and consensus mechanisms to create and store large blocks of transaction data in series. Each block contains the cryptographic hash of the previous block, the corresponding time stamp, and the transaction data, usually expressed as a hash value calculated by the Merkle tree algorithm, which makes the content of the block difficult to tamper with. A decentralized ledger linked with blockchain technology allows both parties to efficiently record transactions and check them permanently. The most prominent application of blockchain technology is currently digital currency, such as Bitcoin. The essence of payment is to “increase the amount reduced in account A to account B”. If one has a public ledger that records all transactions to date for all accounts, then for any account, one can calculate the amount of money it currently has. Blockchain is a public ledger for just this purpose, and it stores all the transactions. In the Bitcoin system, a Bitcoin address is equivalent to an account, and the number of Bitcoins is equivalent to the amount of money.

### 8.1. The Blockchain Architecture

The overall architecture of the blockchain comprises a data layer, network layer, consensus layer, and contract layer [218].

#### 8.1.1. Data Layer

**Figure 14:**
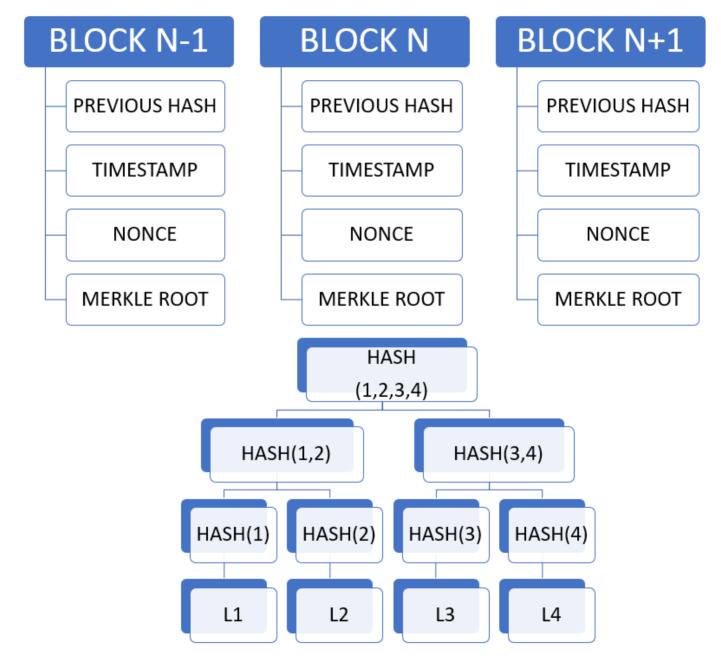
Blockchain data layer.

The structure of the data layer is shown in Figure 14. The block records the quantity of the current transaction and transaction history (L1) and is encrypted by hash to form a database called the Merkle tree. The part of the chain contains the previous hash, timestamp, nonce, Merkle root, and other information. The previous hash provides a link to the previous block. When data are written, a timestamp is generated as proof of data existence, which helps to form the basis for data non-tampering and non-falsification. A nonce is a random number generated whenever a block is created. We can think of it as a key. Thus, the hash value of the current block is equal to the hash value of the previous block concatenated with the nonce. Merkle root serves as the root node of the data.

**Figure 15:**
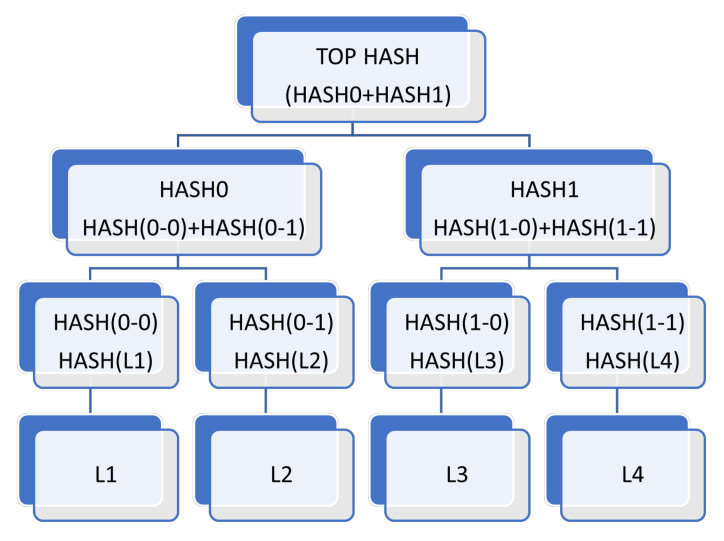
Merkle tree.

A Merkle tree [219] (Figure 15) is also commonly referred to as a hash tree, namely a tree that stores hash values. The leaves of a Merkle tree are the hash values of data blocks (e.g., files or collections of files). Non-leaf nodes are the hashes of strings formed from the concatenation of their child nodes. For instance, Hash 0–0 and Hash 0–1 are the hash values of data blocks L1 and L2, respectively. Hash 0 is the hash value obtained by combining the hash 0–0 and 0–1. The hash algorithm is an algorithm that converts data of arbitrary length to data of fixed length. For data integrity checking, the simplest way is to perform a hash operation on the data to obtained a fixed-length hash value and then publish the obtained hash value on the Internet. The user can download the data, perform the hash calculation again, and compare the result with the hash value published on the Internet. If the two hash values are equal, the downloaded data are considered uncorrupted.

Hash has the following features:

- Determinism: there is a one-to-one correspondence between the data and the hash value, i.e., the same data will get the same hash value.
- Irreversibility: the process of hashing is irreversible, i.e., the data can obtain the hash value through hashing, but the original data cannot be deduced from the hash value, thus ensuring the privacy and security of the data.
- Uniformity: the hash value of the data of any length has a fixed length, which can compress the data and reduce the required storage. This is convenient for later comparison and verification.

When it comes to the verification of a huge amount of data, it takes a lot of memory to store and secure it, which is problematic. Merkle tree is the foundation of blockchain technology, making it easy to find out what data have changed in a large amount of data. Merkle tree is used in both Bitcoin and Ethereum. Merkle tree is essentially a tree-like data structure composed of hashes. It inherits the functions of hashes to ensure data security and privacy and verify data accuracy and integrity, mainly for peer-to-peer downloads. It is difficult to guarantee that each node in these decentralized systems will provide true and trustworthy data, and it is also challenging to avoid data loss and corruption during transmission. Hence, we need to introduce a data encryption and verification mechanisms. Merkle tree is a tree formed by dividing data into many small pieces and hashing them. If we do not split the data and encrypt it directly, it would be hard to determine the source when there is a problem with data validation.

#### 8.1.2. Network Layer

**Figure 16:**
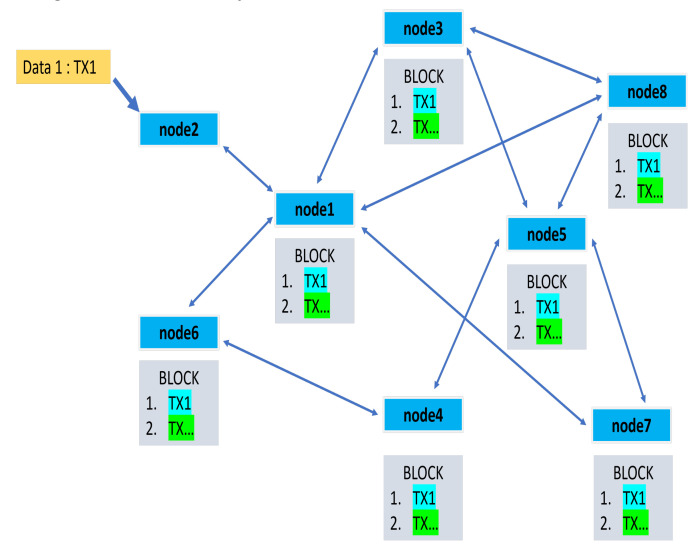
Blockchain P2P network architecture.

Blockchain adopts a P2P (peer-to-peer) network architecture, which abandons the original central server and instead relies on groups of users (peers) as decentralized servers. In the P2P architecture, each node has the functionality of a server. Figure 16 illustrates the P2P network. Data1 is the newly generated data and TX1 is its content. The data are added to node2 and sent to every other node through the P2P network. After receiving data from neighboring nodes, a node first verifies the validity of the data. If the data are valid, a storage pool is created for the new data in the order it is received to temporarily store the valid data that has not yet been recorded in the block while continuing to forward it to neighboring nodes. If the data are invalid, it is immediately discarded, thus ensuring that invalid data will not continue to propagate in the blockchain network. The data of the whole blockchain network is stored on all nodes of the decentralized system simultaneously. Even if some nodes fail or have validation errors, there are still functioning nodes, and the data of the main blockchain chain can be fully restored without affecting the recording and updating of the data of subsequent blocks. This completely decentralized model provides a high level of security.

#### 8.1.3. Consensus Layer

There are two main types of consensus, as described in the following.

- Proof of Work (PoW) Consensus: the core idea is to ensure data consistency and consensus security by introducing the competition of arithmetic power of distributed nodes. By calculating random numbers, the hash value of the function is continuously sent to the nodes until the final answer is within the range set by the algorithm to successfully pack the blocks and continuously synchronize the data in the chain to other nodes to reach consensus [220]. The advantage of the PoW consensus mechanism is to ensure the security and decentralization of the system by competing for computing power.
- PoS (Proof of Stake) Consensus: PoS consensus essentially uses proof-of-stake to replace hash-based proof-of-work in PoW. In PoS, the node with the highest stake in the system rather than the highest power is given the right to keep track of the blocks. The advantage of PoS is to transform arithmetic competition into equity competition, which not only saves arithmetic power but also prevents nodes from launching malicious attacks. At the same time, it makes all nodes responsible for maintaining the safe and stable operation of the blockchain to protect their own interests [221].

#### 8.1.4. Contract Layer

Smart contract is a core component of a blockchain. A smart contract is an event-driven, stateful computer program running on a replicable shared blockchain ledger. It enables active or passive data processing, receiving, storing, and sending values. It controls and manages various types of smart assets on the chain. Smart contracts are signed by all parties, attached to the blockchain data in the form of program code, and then recorded in a specific block of the blockchain after propagation by a P2P network and verification by nodes. The blockchain can monitor the status of smart contracts in real time and activate and execute them by verifying external data sources and confirming that certain trigger conditions are met [222].

### 8.2. Main Security Issues of Blockchain

Blockchain may be vulnerable to several types of attacks.

#### 8.2.1. DNS Attack

Domain name system (DNS) attack [223] is a common type of attack that existed before blockchain. When a new node joins a blockchain, it first needs to access the DNS to obtain information about other nodes and decide which network it wants to join. If a hacker attacks the DNS and implants malicious node information in it, the node may be added to a malicious block created by the hacker, Eventually, a group of malicious nodes constructed by the hacker will provide incorrect blocks to the blockchain. One of the possible solutions for DNS attacks is the reputation system [224]. The reputation system is a way to express the reputation of each node through the contribution of nodes to the whole network, including growth, security, and stability, by giving opinions and ratings to each other by a number of entities. Using a reputation system to select more reliable nodes can prevent a node from being directed to malicious nodes. The reputation system makes it easier to quantify trustworthiness. As reputation becomes more visible, the system also becomes more effective.

#### 8.2.2. Border Gateway Protocol (BGP) Hijacking

BGP attains full/light node control by hijacking routers. Such attacks can delay block propagation on a blockchain by up to 20 min [225]. A full node has the complete blockchain ledger, along with the ability to independently verify the authenticity of transactions. A light node is specifically defined as a node that does not store or maintain a complete copy of the blockchain, but only stores a minimal amount of state for sending or transmitting transaction messages.

#### 8.2.3. Distributed Denial of Service Attacks (DDoS)

Conceptually, a DDoS attack involves an excessive number of accesses to a target computer or system, causing it to run out of resources and forcing a temporary disruption of service. The attack affects regular user access or normal user transaction verification. Blockchain is a mechanism of “whoever has the computing power always prevails”, hackers paralyze the computing power of other miners through DDoS to increase the proportion of computing power they hold, which is the main purpose of this attack method. Several types of DDoS attack are described in.

Possible solutions include flow cleaning and augmented resource computing [226]. Flow cleaning performs the real-time monitoring of data traffic to accurately identify abnormal attack traffic. It then cleans off the abnormal traffic without affecting the regular business traffic, thereby achieving flow restriction of the server. Augmented resource computing means that when a DDoS attacker tries to flood the system, the system can temporarily increase computing resources to allow the server to continue operation without being paralyzed and immediately take countermeasures against DDoS to minimize the damage.

#### 8.2.4. GPS Spoofing Attacks

GPS spoofing is a common attack method to disrupt positioning. This type of attack works by sending fake signals to terminals equipped with GPS receivers and has happened in smartphones, drones, yachts, and Tesla cars. In particular, AVs need to use sensors to sense the surroundings, measure the distance, and plan paths based on the data collected to facilitate self-driving. Specifically, ultrasonic sensors are responsible for measuring short distances, high-definition cameras identify road signs and vehicle distances, and radar generates three-dimensional maps. If these sensors are attacked to generate incorrect input, they will interfere with the navigation system and make incorrect judgments.

One possible solution is a signature-based mechanism [227]. When the receiver is not sure whether the received data are from the original sender, the digital signature mechanism can ensure the accuracy of the data received and effectively prevent the interception and tampering of the data.

#### 8.2.5. Sybil Attacks

Malicious participants can create multiple identities. Once enough fake identities are created, they can defeat trusted nodes on the network with multiple votes and can refuse to receive or transmit blocks, thus preventing other users from accessing the network. In a large-scale Sybil attack, the order of transactions can be easily changed or even reversed to prevent them from being confirmed, leading to problems such as double payments [228]. Possible mitigations are identity authentication and proof of work (PoW). The nodes are authenticated by a unified third-party organization, such as oracles [229], which is a third-party trusted organization. However, this will sacrifice some of the anonymity of the authenticated nodes. If the participating P2P nodes can accept the trade-off, then this is indeed a viable solution. Proof-of-work mechanisms use computational power to prove that a node is an authentic node, which can substantially increase the cost of an attack.

### 8.3. Blockchain in Autonomous Vehicles

Blockchain can be used in a variety of ways in AVs.

1. Self-driving technology: the data obtained from vehicle sensors are stored in the blockchain so that all parties can monitor and share the safety information of the vehicle and the way the car owner uses the car in a more stringent way, increasing the transparency of information and reducing the risk of data theft.
2. Telematics: telematics uses blockchain technology to provide secure and robust data distribution and interoperability among multiple self-driving vehicles and other entities, such as regional authorities and public facilities.

Considerations in the application of blockchain technology in autonomous vehicles include:

1. Vehicle safety: sensors on the vehicles are used to detect speeding, alert or even control the vehicle, or detect the continuous drifting of the vehicle to prevent driving negligence and reduce accidents. Some insurance companies have also proposed using the sensor data stored on the blockchain to assess driving habits that form the basis for insurance premiums.
2. Accident management: automatic positioning and emergency assistance in an accident are the most important functions of accident management. With an onboard computer, wireless communication technology, and global satellite positioning technology, we can send a help signal to rescue organizations in the first instance when an accident occurs. Blockchain can supply relevant information and help determine the exact location of the vehicle and the severity of the accident, which brings great help to the rescue work in a race against time.
3. Vehicle monitoring: vehicle monitoring incorporates global satellite positioning technology and wireless communication technology. It integrates various value-added services such as command dispatch, target tracking, emergency alarm, and information release into one blockchain. Through the non-tamperable feature of the blockchain system, the route, fatigue driving, overloading, and emergency alarm can be monitored, effectively improving the credibility of information.

### 8.4. Challenges of Blockchain Applications in Autonomous Vehicles

Currently, several challenges are faced by blockchain for applications in AVs.

- Because of anonymity, it is impossible to confirm the accuracy of data sources.
- It is not yet possible to achieve a situation in which security, scale, and execution efficiency are all satisfied.
- The technical impact and popularity of the technology are not yet high.

Blockchain data are anonymous and there is literally no way to identify which vehicle the data belong to. Although decentralized identifier (DID) technology is available to obtain IDs for vehicles, it is not yet widespread enough to establish a comprehensive system to manage them. The system is not only complex but also difficult to manage in the future.

Moreover, in self-driving vehicular networks, vehicles are highly mobile. Therefore, there are concerns regarding the performance of blockchain networks. Currently, there lacks a blockchain technology framework for the mobile environment of self-driving vehicular networks [230]. Additionally, the smart contract specification is not widely available. The data on the sensors in a self-driving vehicle or the vehicular networking technologies V2I (vehicle-to-infrastructure, vehicle-to-road system), V2N (vehicle-to-network), V2V (vehicle-to-vehicle), V2P (vehicle-to-pedestrian), and V2D (vehicle-to-device) need to be regulated as a smart contract to protect their respective rights before they are added to the blockchain system. With the advent of the big data era, self-driving cars are certainly no exception. The enormous amount of data produced by various applications and how blockchain technology works in the infrastructure with high computing requirements are also challenges.

## 9. Future Research Directions

The lingering challenge in the autonomous vehicle sector is spearheading future research to make the architecture more robust for commercialization. We have attempted to identify some of the areas where extensive research is being or will be carried out in the near future [79,230]. It is worth noting that the following list is increasing, with new doors opening every day in ITSs in general and in the autonomous vehicle domain in particular.

1. *Emergence of high-definition (HD) maps with big data and HPC*: The rapid improvement in sensor technology has strengthened the perception and localization functions of AVs in diverse environments. With the likes of LiDAR, Radar, efficient cameras, and GNSS, among others, autonomous vehicles are getting better each day at perceiving their surroundings as efficiently as humans. The big data generated from the fusion of the myriad sensors on board can improve the performance of AVs to a great extent, even in complicated and unpredictable terrain [134]. Besides big data, the emergence of high-performance computing (HPC) devices along with other relevant infrastructure sensor measurement information can pave the way for the formulation of real-time maps. HD maps will serve as the key input of the complex AI algorithms to be implemented in the AV architecture and interact with AVs by reflecting real-world scenarios. HD maps will help improve perception, localization, planning, and decision making. HD map startups have resorted to computer vision technology and crowd-sourcing to develop real-time HD maps of the localities, with perks offered to users who will use their apps to record data that will later be used to make HD maps. By relying on HD maps, AVs can choose to archive the inputs of traffic signals and other vehicles and instead, use DL or RL approaches to realize direct perception and decision making. This will help in diminishing the software and hardware costs [82]. Based on the maps, AVs can easily navigate and choose the less congested and shorter paths to a parking space among the ones shown on the map and drive towards it, guided by the markings on the map.
2. *Risk assessment*: For AVs to reach their destinations safely, they need to assess and predict the paths of other entities (other vehicles, pedestrians, human drivers, etc.) on the road alongside them. Risk assessment is vital to averting collisions on the road, which still occur today due to misplaced perception and decision making. Most risk assessment approaches focused on trajectory prediction and the subsequent detection of a collision which is computationally exhaustive and time-consuming. Later, a better approach included going for trajectory calculation and collision prediction only if a dangerous maneuver or bad traffic condition was detected [231]. An autonomous vehicle on the road is no longer treated as a single entity but as part of a wider traffic system. This shift in perspective will enable future studies to account for the surrounding traffic system complexity in risk assessment by exploring traffic engineering approaches such as the detection of crash precursors and network-level crash prediction. Since an AV also interacts with other road participants such as human drivers, pedestrians, cyclists, and so on, a planning algorithm is needed to explore the reasoning ability of human behavioral models, which will be used in trajectory calculations or predicting the actions of fellow road participants at a crossing. Behavioral models can be provided for every maneuver that the vehicle undertakes to classify it as normal or not [232,233].
  The inherently uncertain nature of trajectory prediction is driving researchers in the behavior prediction community to see beyond single mean average precision (MAP) prediction and generate methods related to probabilistic predictions [234,235,236]. This involves learning the joint distributions of future states of all the concerning agents, based on their past trajectories and a few specific variables. Since learning such a distribution can be a tedious and demanding job, current works explore a wide range of simplified representations of probabilistic predictions. Some instances involve training a conditional variable auto-encoder (CVAE) to generate samples of possible future trajectories [237], whereas others use generative adversarial networks (GANs) to generate multiple trajectories with attached probabilities [238]. Nonetheless, others train a deep neural network (DNN) to generate a grid-based map with probabilities assigned to each cell. The recent trend involves learning Gaussian mixture models (GMMs) as representatives of vehicle positions [239,240]. Since real-time latency constraints pose a major barrier in probabilistic predictions for autonomous vehicle risk assessment, the authors in [241,242] proposed fast, non-sampling-based methods for the risk assessment of trajectories in both Gaussian and non-Gaussian position and control models of other agents on the road. However, existing deep-learning-based methods often fail when faced with complex real-life scenarios and are not transferable. Thus, the authors in [243] proposed a novel approach using “scene graphs” as intermediate representations. Such a data-driven approach includes a multi-relation graph convolution network, a long short-term memory (LSTM) network and attention layers to imitate the subjective risk of driving maneuvers.
3. *Enhanced simulation testbed with AR/VR*: The training and testing of various AI algorithms for AVs are time-consuming. That is why many existing approaches rely on simulation platforms such as MATLAB and CarSim to emulate AVs and their surrounding environments. However, such simulation testbeds lack the crucial part involving interaction with the traffic agents present around the vehicle, such as other vehicles, crossing pedestrians, cyclists, and so on. Consequently, the AI models in AVs miss out on training in realistic scenarios and lack the capability to evolve and react in a better way to unprecedented situations. A promising solution is training the designed AI algorithms using augmented reality (AR)/virtual reality (VR) to emulate real human behaviors in estimating the potential safety issues of AVs [244,245]. Pedestrians can wear VR/AR devices to help researchers simulate a realistic situation on a road through which an AV will drive. Their behavioral data will be collected and used for training/testing AI. This will develop extensive scenarios and provide rich data for training the AI algorithms, enable them to obtain acclimated with people’s behavior, and evolve faster to deal with all possible situations on the road prior to field implementations. Mixed reality (MR) prototyping with AR and VR provides a secure testing platform for AI models which are yet to be perfected and can be specially used for testing in risky situations without jeopardizing humans. This can foster the use of reinforcement learning (RL) approaches to interact with actual pedestrians wearing AR/VR devices and imitate a human driver’s decision making, optimize the steering angles accordingly and avert collisions.
4. *Green energy solutions*: environmental concerns have forced vehicle makers and researchers to explore and develop alternative, greener sources of energy for everything. Although such alternatives as solar, wind, hydropower, geothermal, biomass, and biofuel energy have their own constraints (such as production, storage, and distribution) and are yet to achieve the efficiency delivered by fossil fuel-based energy resources such as coal and petroleum, they are steadily improving as a viable alternative to counter global warming, depletion of non-renewable resources, and pollution. In light of the current environmental trends, researchers must explore renewable energy sources as backup energy systems for AVs. It is high time to explore such green energy solutions in depth. The role of blockchain technology in energy production and in the consumption cycle has the potential to unlock several doors for further research. The authors in [246] deliberated on how the lack of a proper management platform for vehicular network computing is severely thwarting the development of the system. The use of green energy and the communication latency between jobs and block creation is part of an integrated optimization strategy. Integrating green energy solutions and system performance with AVs and vehicular networks will help ameliorate present and future costs and improve efficiency.
  Electric autonomous vehicles are a rising solution to tackle traffic congestion and air pollution problems in futuristic smart cities. However, the presence of power-hungry computing units such as GPUs that fuse and process data from an array of sensors on-board produces more than 12 GB of data per minute and consumes nearly 2.5 KWh of energy [247]. In such a case, a fully charged AV will fall behind the standard mileage [248]. Hence, charging electric AVs is of paramount importance. The authors in [249] proposed leveraging solar energy to power autonomous vehicles through efficient charging stations and extensive solar-harvesting rooftops. This burdens the service provider with additional responsibilities to offer a better coverage of charging stations, guided route planning, and operation management. Ensuring a holistic solution to the charging problem will assist the AVs with efficient algorithms at the infrastructure backend to offer better decision making.
5. *Improvement of QoS*: more in-depth studies on QoS and ways to improve it without compromising security aspects are needed. Important questions need to be asked while improving the performance of AVs, such as which algorithms will provide both security and real-time responses to autonomous vehicles. Or, how the amount of variation in the renewable energy utilization rate and QoS score will be affected by the number of blocks with differing capacities. Details related to blockchain need to be standardized and designed exclusively for AV architecture, such as block size, workload, network size, node configuration, and programmable application interfaces. The contributions in [250,251,252,253,254,255] can be extended to perform in-depth security analysis concerning attacks such as DoS, DDoS, ballot stuffing, etc. It should also involve detailed performance analysis, such as trust and network device’s reputation score variation with time, response time, and smart contract execution time for vehicular ad hoc networks. The enhancement of system performance with the implementation of various models should be compared based on simulation results. The stiff comparison should be based on four benchmark aspects, namely throughput (S), packet loss rate (PLR), packet delivery ratio (PDR), and average delivery latency (DL).
  Recent promising work in [256] proposed a novel optimization model to find the best position of neighboring vehicles in a fleet to efficiently communicate with AVs or relay messages between AVs. The evaluation of the QoS parameter (based on reliability, effectiveness, and optimum transmission distance between V2V and V2I in a network) will be the deciding factor in whether an AV will communicate directly with the destination or via cooperative communication. This work can be further extended to form an adaptive intelligent transportation system where the AV will decide where to communicate with, by determining the optimum transmission distance to achieve the required QoS in a dynamic way. Future works on this should include multiple relay nodes to gain significant system enhancement. More simulation and investigation are needed to assess its practical applicability as several aspects can degrade the QoS in real-life dynamic environments.
6. *Smart contracts*: Smart contracts are digital, self-executing programs stored in blockchain that contain the delicate information controlling the transfer of digital assets among parties after the fulfillment of certain conditions. The details and permissions are set up by their creators in executable lines of code, implementing the security of blockchain, and can be executed without the need for trusted intermediaries [257]. Being embedded in blockchain makes smart contracts decentralized, immutable, inexpensive, and transparent. This spurred researchers to think about designing smart contracts to suit AV architecture. The need for such a digital contract for AVs arises from the promise of having fast, responsible, and autonomous transactions. By accessing the events stored by a smart contract in its memory location, the design, implementation, and analysis can be looked into and extended to cover autonomous vehicles and systems. The authors in [252,258,259] discussed smart contracts in which the reputation of blockchain-integrated vehicular networks is built through trust management. Smart contracts execute transactions only after receiving a good reputation rating from the trust management system. As such, the security of AVs will not be compromised by attackers. Many recent works focused on using smart contracts to guarantee the authenticity and integrity of new firmware updates of AVs [260]. When it comes to peer-to-peer (P2P) energy trading for autonomous and other vehicles, blockchain and smart contracts together can provide decentralized yet secure energy trading between producers and consumers (energy users) and encourage the conservation of energy [261].

## 10. Conclusions

Autonomous vehicles are a milestone in the latest line of technological developments that serve as an important indicator of technological evolution. They are expected to reduce traffic accidents and improve road safety by eliminating dangerous driving behaviors such as fatigue driving. In this paper, we extensively examined the enabling technologies that are critical to the realization of autonomous vehicles, pointing out the connection between each of the technologies and the development of AVs. IoV and IoAV represent special types of IoT applied to vehicles. As a combination of AI and edge computing, edge intelligence will empower vehicles to make smart decisions in the face of urgency. Explainable AI will further enhance the credibility of the reasoning of AI behind the decision. Moreover, 5G and 6G mobile communication will make the transfer of huge amounts of data that are crucial to the operation and control of AVs more efficient and robust. Finally, blockchain and related technologies are essential for protecting AVs from potential security and privacy threats. In addition to the crucial role of individual technologies, we also elaborated on the challenges that need to be overcome and the deficiencies that need to be strengthened. All of the technologies are still evolving and advancing at a fast pace. We identified several directions for future research efforts. These should provide insights and spur research to expedite the development and maturity of intelligent autonomous vehicles.

## Figures and Tables

**Table 1:** Disadvantages of sensors used in autonomous vehicles.

Sensor Type	Disadvantages	Source
GNSS	The update rate is too slow for the real-time requirements of AV Cannot work accurately in closed spaces (such as tunnels and crowded city streets) as it requires an unhindered view of the sky Exposure to and interference from other waves in different radio frequency spectra hampers performance Receivers may receive multiple signals reflected by surrounding objects (buildings, walls, etc.), introducing unwanted noise (multi-path errors) Prone to cyberattacks, deliberate jamming, and spoofing	[12,24]
IMU	Accuracy degrades over time Constantly rounds of small fractions of errors in its measurements (relative to itself), which accumulate over time, leading to significant errors, known as Drift	[12]
LiDAR	Initial and maintenance costs are very high Performance degrades significantly in bad weather such as fog, rain, and snow since it uses visible lasers for object detection and distance measurement It is the most power-hungry sensor, significantly reducing the AV’s driving range External elements and architectural features create occlusions that may lead to partial data collection or inaccurate measurements since it is a line-of-sight technology	[25,26]
Camera	Inconsistent performance across different illumination conditions (for example, it can be blinded by strong light) Performance degrades in bad weather such as snow, rain, and fog Poor depth perception and low range Requires high processing power for the large volume of data generated	[27,28]
RADAR	Increasing mutual interference among automotive radars leads to inaccurate perception The generated point cloud after reflection of radio waves gives minimal information about the spatial dimensions of objects	[14,29]

## Data Availability

Not applicable.

## Abbreviations

The following abbreviations are used in this manuscript:

**Acronym**	**Definition**
3GPP	Third-Generation Partnership Project
5G	Fifth-Generation (Mobile Telecommunications Technology)
6G	Sixth-Generation (Mobile Telecommunications Technology)
ACC	Adaptive Cruise Control
ADAS	Advanced Driver Assistance System
AEB	Automated Emergency Braking
AI	Artificial Intelligence
AGI	Artificial General Intelligence
AM	Additive Manufacturing
ANI	Artificial Narrow Intelligence
ANN	Artificial Neural Network
AR	Augmented Reality
ASI	Artificial Super Intelligence
AV	Autonomous Vehicle
AUV	Autonomous Underwater Vehicle
BGP	Border Gateway Protocol
BO	Bayesian Optimization
BS	Base Station
BSM	Blind-Spot Monitoring
CC	Common Criteria
CNN	Convolutional Neural Network
CPS	Cyber-Physical Systems
CVAE	Conditional Variable Auto Encoder
D2D	Device-to-Device
DDT	Dynamic Driving Task
DDoS	Distributed Denial of Service
DIDs	Decentralized Identifiers
DL	Deep Learning or Delivery Latency in some contexts
DM	Driver Monitoring
DNN	Deep Neural Network
DNS	Domain Name Server
DoS	Denial of Service
DSRC	Dedicated Short-Range Communications
EI	Edge Intelligence
ECU	Engine Control Unit
EKF	Extended Kalman Filter
ETSI	European Telecommunications Standards Institute
FPGA	Field-Programmable Gate Array
FW	Feature Weights
GA	Genetic Algorithm
GAN	Generative Adversarial Network
GMM	Gaussian Mixture Model
GNSS	Global Navigation Satellite System
GPS	Global Positioning system
GPU	Graphical Processing Unit
HARQ	Hybrid Automatic Repeat Request
HD	High Definition
HMM	Hidden Markov Model
HPC	High Performance Computing
ICT	Information and Communication Technology
IMU	Inertial Measurement Unit
IoT	Internet of Things
IoAV	Internet of Autonomous Vehicles
IoV	Internet of Vehicles
IT	Information Technology
ITS	Intelligent Transportation System
IVSS	Intelligent Video Surveillance System
KWh	Kilo-Watt Hour
LAN	Local Area Network
LDPC	Low-Density Parity Check
LIDAR	Light Detection and Ranging
LR	Linear Regression
LSTM	Long Short-Term Memory
LTE	Long-Term Evolution
MAC	Medium Access Control
MCC	Mobile Cloud Computing
MEC	Mobile Edge Computing
ML	Machine Learning
M2M	Machine-to-Machine
MIMO	Multiple-Input–Multiple-Output
MITM	Man in the Middle
MQTT	Message Queuing Telemetry Transport
MR	Mixed Reality
NEMO	Network Mobility
NFV	Network Function Virtualization
NIST	National Institute of Standards and Technology
NR	New Radio
NS	Network Simulation
OBU	On-Board Unit
QoS	Quality of Service
PDR	Packet Delivery Ratio
PEBS	Predictive Emergency Braking Systems
PHY	Physical layer
PLR	Packet Loss Rate
P2P	Peer-to-Peer
ProSe	Proximity Service
RAC	Routing Area Code
RAN	Radio Access Network
RGB	Red Green Blue
RL	Reinforcement Learning
R2P	Roadside and Personal Device
R2R	Roadside-to-Roadside
RNN	Recurrent Neural Network
RSU	Roadside Units
RTEPC	Real-Time Embedded Personal Computer
SA	Simulated Annealing
SAE	Society of Automotive Engineers
SDN	Software Defined Network
SGD	Stochastic Gradient Descent
SLAM	Simultaneous Localization and Mapping
SUMO	Simulation of Urban Mobility
THz	Tetra-Hertz
TJA	Traffic Jam Assist
UAV	Unmanned Aerial Vehicle
UE	User Equipment
URLLC	Ultra-Reliable Low-Latency Communication
VANET	Vehicular Ad Hoc Network
V2H	Vehicle-to-Human devices
V2I	Vehicle-to-Infrastructure
V2N	Vehicle-to-Network
V2P	Vehicle-to-Personal Device
V2R	Vehicle-to-Roadside
V2V	Vehicle-to-Vehicle
V2X	Vehicle-to-Everything
VLC	Visible Light Communications
VMNO	Virtual Mobile Network Operators
VR	Virtual Reality
WAN	Wide Area Network
WLAN	Wireless Local Area Network
WSN	Wireless Sensor Network
XAI	Explainable AI
